# MUC1 Specific Immune Responses Enhanced by Coadministration of Liposomal DDA/MPLA and Lipoglycopeptide

**DOI:** 10.3389/fchem.2022.814880

**Published:** 2022-02-04

**Authors:** Jing-Jing Du, Shi-Hao Zhou, Zi-Ru Cheng, Wen-Bo Xu, Ru-Yan Zhang, Long-Sheng Wang, Jun Guo

**Affiliations:** ^1^ Hubei Key Laboratory of Kidney Disease Pathogenesis and Intervention, College of Medicine, Hubei Polytechnic University, Huangshi, China; ^2^ Key Laboratory of Pesticide and Chemical Biology of Ministry of Education, Hubei International Scientific and Technological Cooperation Base of Pesticide and Green Synthesis, International Joint Research Center for Intelligent Biosensing Technology and Health, College of Chemistry, Central China Normal University, Wuhan, China; ^3^ Hubei Provincial Key Laboratory of Green Materials for Light Industry, School of Materials and Chemical Engineering, Hubei University of Technology, Wuhan, China

**Keywords:** DDA, MPLA, MUC1, lipoglycopeptide, liposome, cancer vaccine

## Abstract

Mucin 1 (MUC1), a well-known tumor-associated antigen and attractive target for tumor immunotherapy, is overexpressed in most human epithelial adenomas with aberrant glycosylation. However, its low immunogenicity impedes the development of MUC1-targeted antitumor vaccines. In this study, we investigated three liposomal adjuvant systems containing toll-like receptor 4 (TLR4) agonist monophosphoryl lipid A (MPLA) and auxiliary lipids of different charges: cationic lipid dimethyldioctadecylammonium (DDA), neutral lipid distearoylglycerophosphocholine (DSPC) or anionic lipid dioleoylphosphatidylglycerol (DOPG), respectively. ELISA assay evidenced that the positively charged DDA/MPLA liposomes are potent immune activators, which induced remarkable levels of anti-MUC1 antibodies and exhibited robust Th1-biased immune responses. Importantly, the antibodies induced by DDA/MPLA liposomes efficiently recognized and killed MUC1-positive tumor cells through complement-mediated cytotoxicity. In addition, antibody titers in mice immunized with P_2_-MUC1 vaccine were significantly higher than those from mice immunized with P_1_-MUC1 or MUC1 vaccine, which indicated that the lipid conjugated on MUC1 antigen also played important role for immunomodulation. This study suggested that the liposomal DDA/MPLA with lipid-MUC1 is a promising antitumor vaccine, which can be used for the immunotherapy of various epithelial carcinomas represented by breast cancer.

## Introduction

Mucin1 (MUC1), a transmembrane glycoprotein highly overexpressed and aberrantly glycosylated on many tumor tissues including ovarian, breast, pancreatic, prostate and ovarian carcinomas (Hollingsworth and Swanson, 2004; Kufe, 2009; Nath and Mukherjee, 2014; Chen et al., 2021). MUC1 glycoprotein contains a variable number of tandem repeats (VNTRs) region (HGVTSAPDTRPAPGSTAPPA) in its extracellular domain (Gaidzik et al., 2013; Pillai et al., 2015; Li and Li, 2020). With its unique biological features, tumor-associated antigen MUC1 glycoprotein has been considered as one of the favorable targets for the development of cancer immunotherapy (Barratt-Boyes, 1996; Singh and Bandyopadhyay, 2007; Pillai et al., 2015; Dhanisha et al., 2018; Brockhausen and Melamed, 2021). However, the weak immunogenicity of MUC1 limits its development and clinical application (Tang et al., 2008; Tang et al., 2018; Chen et al., 2021). To increase its immunogenicity, co-delivery of immunostimulating components and antigens establish an effective strategy for cancer vaccine (Ingale et al., 2007; Cai et al., 2014; Yin et al., 2017; Wu X. et al., 2018; Wu J.-J. et al., 2018; Supekar et al., 2018; Liu et al., 2021; Wu et al., 2021; Zhu et al., 2021).

TLRs present on diverse cells like macrophages, Dendritic cells (DCs), B cells and natural killer (NK) cells (Adams, 2009; Baxevanis et al., 2013; Li et al., 2017; Gao and Guo, 2018; Owen et al., 2021; Zhou et al., 2022). Monophosphoryl lipid A (MPLA), a TLR4 agonist optimized from *Salmonella minnesota* lipopolysaccharide (LPS), is a promising immunostimulant licensed for use in human vaccines preventing viral infections (Alderson et al., 2006; Vacchelli et al., 2012; Gao and Guo, 2018; Shetab Boushehri and Lamprecht, 2018; Romerio and Peri, 2020). MPLA plays an important role in stimulating the maturation of DCs, inducing the upregulation of major histocompatibility complex (MHC) class I and II molecules, and promoting the migration of DCs to CD4 T cells (Akira and Takeda, 2004). In addition, MPLA is being actively investigated as a potent immunostimulatory adjuvant to cancer vaccines (Cluff et al., 2005; Didierlaurent et al., 2009; Wang et al., 2017; Zhou et al., 2017; Facchini et al., 2021).

**FIGURE 1:**
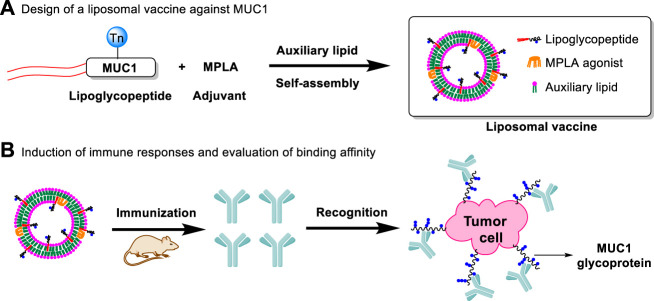
Design of a liposomal vaccine consisting of auxiliary lipids, MUC1 lipoglycopeptides and MPLA adjuvant. **(A)** Design of a liposomal vaccine against MUC1; **(B)** Induction of immune responses and evaluation of binding affinity.

Lipid modification of peptides can promote self-assembly and formation of liposomes, which can enhance their immunogenicity by presenting the multivalent antigens and increasing uptake by antigen presenting cells (APCs) (Eskandari et al., 2017; Aiga et al., 2020). In addition, our previous studies showed that co-delivery of adjuvants and antigens via liposomes significantly increased the immunogenicity of antigen (Du et al., 2019). As liposomal adjuvant, MPLA can also effectively participate in the formation of liposomes and enhance immune responses (Figure 1).

Based on the above considerations, we developed liposomal vaccines using the amphiphilic lipidated MUC1 glycopeptides as target antigens and TLR4 agonist MPLA as immunoadjuvant. Considering that the negative charge of the MPLA adjuvant may affect the assembly of liposomes (Brandt et al., 2000; Korsholm et al., 2010; Wang et al., 2013), we designed three commonly applied auxiliary lipids of different charges including cationic lipid dimethyldioctadecylammonium (DDA) (Hilgers and Snippe, 1992; Korsholm et al., 2007), neutral lipid distearoylglycerophosphocholine (DSPC) and anionic lipid dioleoylphosphatidylglycerol (DOPG), respectively. DDA could efficiently form a kind of cationic liposome, which facilitated the antigen presentation and further induced Th1-biased immune responses (Qu et al., 2018). Phospholipid DSPC (Nakamura et al., 2015) or DOPG (Yanasarn et al., 2011) is used as the key component in the formation of liposomes. Its stability is affected by phospholipid charge, which can influence drug delivery efficiency. The DDA cationic liposome-forming lipid has been reported to enhance the antigen uptake and presentation to T cells as a potent adjuvant (Yu et al., 2012; Wang et al., 2013). In addition, in order to determine the best lipid anchor, four MUC1 glycopeptides were constructed: MUC1, P_1_-MUC1, P_2_-MUC1 and P_3_-MUC1, then the structure-activity relationship between the lipid-tailed MUC1 and the liposomal adjuvant system was studied.

## Results and Discussion

### Chemical Synthesis of MUC1 Glycopeptide and Lipoglycopeptides

**SCHEME 1:**
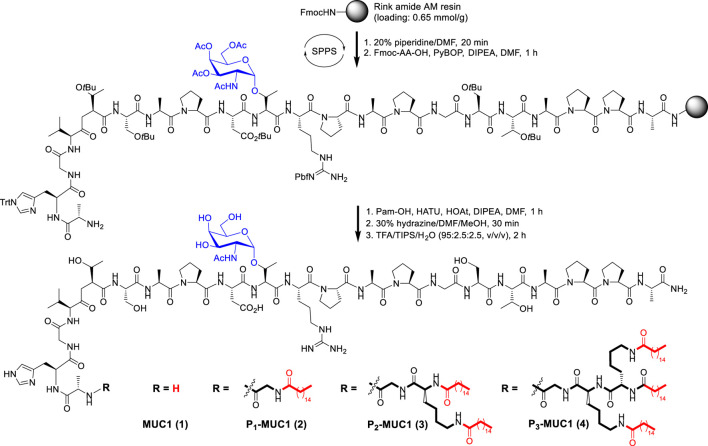
Synthesis of MUC1 glycopeptide and lipoglycopeptides by solid phase peptide synthesis (SPPS).

The resin-bound peptide MUC1 with Tn glycosylation on the PDTRP motif was synthesized via the solid phase methodology using Fmoc strategy (Scheme 1 and Supplementary Schemes S1–S4). Then the palmitic acid was directly conjugated to the resin-bound peptide as described previously (Du et al., 2019). After deprotection and work up, the MUC1 glycopeptide and lipoglycopeptides were isolated in yields of 10–35% and characterized by high performance liquid chromatography (HPLC) and ESI mass spectrometry (Supplementary Figures S6–S13).

### Design and Preparation of Vaccine Candidates

**FIGURE 2:**
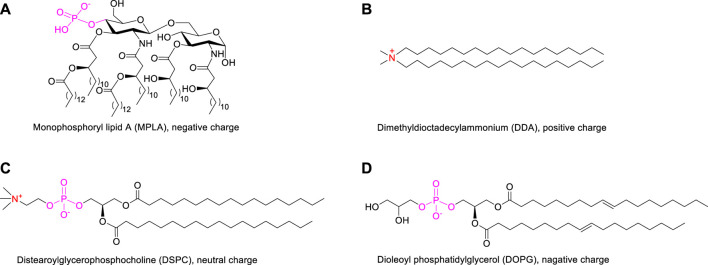
The structures of MPLA adjuvant **(A)** and auxiliary lipids including cationic DDA **(B)**, neutral DSPC **(C)** and anionic DOPG **(D)**.

The design of a liposomal adjuvant based on MPLA and auxiliary lipids of different charges was described in Supplementary Table S1. In this strategy, two issues are mainly discussed: 1) the interaction between auxiliary lipids of different charges and the adjuvant MPLA (Figure 2); 2) the influence of different numbers of lipid chain on the immune activity of vaccines. Liposomes composed of antigens, adjuvant and auxiliary lipids were produced using the lipid-film hydration method (Du et al., 2019). The mole ratio of MUC1 antigen, agonist (MPLA), auxiliary lipid was maintained at 1:1:8 (Karlsen et al., 2014). Subsequently, in order to compare the effect of different auxiliary lipids on vaccine efficacy, positive charged DDA, neutral DSPC and negatively charged DOPG were introduced. Finally, to explore the influence of lipid modification, equimolar amounts of MUC1 glycopeptide (10 nmol, 22 μg), Pam(P_1_)-MUC1 (10 nmol, 25 μg), Pam_2_(P_2_)-MUC1 (10 nmol, 28 μg) and Pam_3_(P_3_)-MUC1 (10 nmol, 32 μg) were employed in the vaccine design. After hydration of a lipid film that contained all components in a 10 mM Tris buffer (pH 7.4), the liposomes were prepared by ultrasound for 20 min.

### Immunization of Mice

Female BALB/c mice (aged 6–8 weeks) were inoculated on day 0, and again on days 14 and 28. Two weeks after each immunization, sera were collected for immune activity assessment (Supplementary Scheme S6). All animal studies were carried out according to National Institute of Health and institutional guidelines. During the immunization period, no weight loss and other abnormal physiological phenomena (such as changes in hair, behavior and appetite) (Supplementary Figure S5) were observed in mice.

### Characterization of Vaccines

**FIGURE 3:**
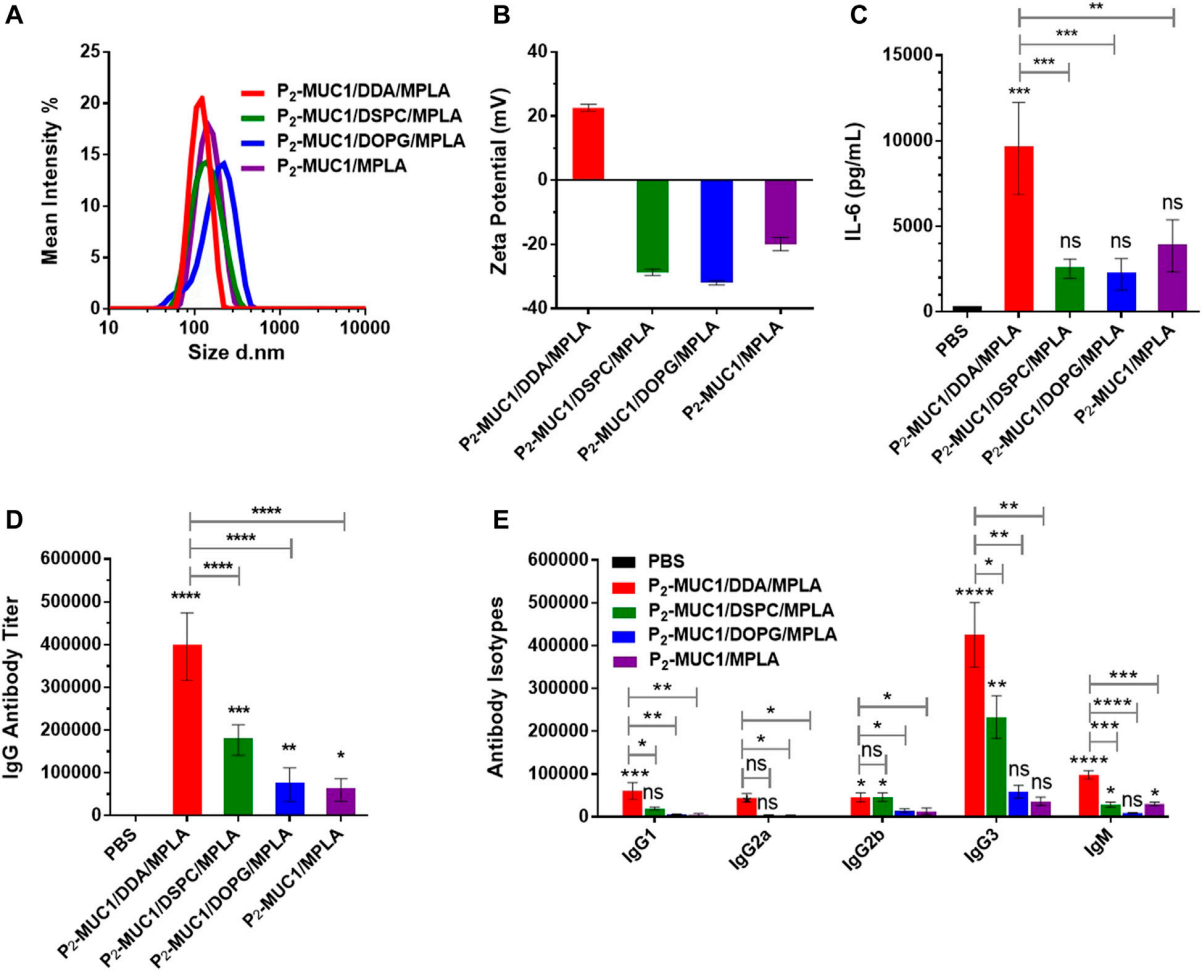
DLS **(A)** and zeta potential **(B)** results for vaccines. Values are presented as the mean ± SD (*n* = 3). The secretions of cytokines **(C)**, MUC1-specific IgG antibody **(D)** and antibody isotypes **(E)** were detected by enzyme-linked immunosorbent assay (ELISA) (*n* = 5). Control: phosphate-buffered saline (PBS). ****, *p* < .0001, ***, *p* < .001, **, *p* < .01, and *, *p* < .05 compared with the control group by one-way analysis of variance (ANOVA) with the Tukey’s HSD test. Comparisons between different groups were also conducted by ANOVA using Tukey’s HSD test. Data represent the mean ± SD of five mice from three separate experiments.

As shown in Figure 3A, the dynamic light scattering (DLS) showed that these liposomes have homogeneous particle sizes with diameters of approximately 100 nm, which may facilitate transport to the lymph nodes (Jiang et al., 2017; Zhang et al., 2018). In addition, zeta potential showed that liposomal DDA/MPLA along with P_2_-MUC1 antigen had a higher value of positive zeta potential than other liposomes (DSPC/MPLA or DOPG/MPLA) (Figure 3B). The charge of liposomes is mainly caused by a combination of auxiliary lipids and MPLA. Due to the mole ratio of DDA and MPLA at 8:1, the cationic DDA makes its corresponding liposome positively charged.

### Liposomal DDA/MPLA Induced a Stronger Immune Response

To assess the interaction between MPLA agonist and auxiliary lipids of different charges, the secretion level of IL-6 after the first immunization was evaluated (Figure 3C). The results showed that all groups containing MPLA agonist produced high levels of IL-6 compared to the control group. This rapid production of cytokines indicated that the TLR signaling pathways were activated in mice immunized with these vaccines. Interestingly, IL-6 cytokines induced by the co-administration of cationic DDA and MPLA was higher than those induced by DSPC/MPLA and DOPG/MPLA. This may be attributed to the better electrostatic attraction between the positively charged DDA and the negatively charged MPLA agonist (Hilgers and Snippe, 1992; Korsholm et al., 2007), which increased uptake of liposomes by APCs.

As DDA/MPLA showed a great ability to induce cytokine secretion, we next explored its potential in stimulating antibody responses *in vivo*. The MUC1-specific IgG antibody titers in sera were measured by enzyme-linked immunosorbent assay (ELISA). Compared with P_2_-MUC1/DSPC/MPLA and P_2_-MUC1/DOPG/MPLA liposomal vaccines, the P_2_-MUC1/DDA/MPLA liposomal vaccine induced higher anti-MUC1 IgG antibody titers (Figure 3D, 2-fold higher than P_2_-MUC1/DSPC/MPLA, 5-fold higher than P_2_-MUC1/DOPG/MPLA). In terms of antibody isotypes, IgG3 titers were greatly increased in mice immunized with DDA/MPLA/P_2_-MUC1 (Figure 3E), which suggested that DDA/MPLA liposome could promote antigen deposition at the injection site and induce prominent Th1-biased response (Henriksen-Lacey et al., 2011). In addition, P_2_-MUC1/DDA/MPLA liposomal vaccine induced higher levels of IgG antibody titers than P_2_-MUC1/MPLA without any auxiliary lipids (6-fold).

**FIGURE 4:**
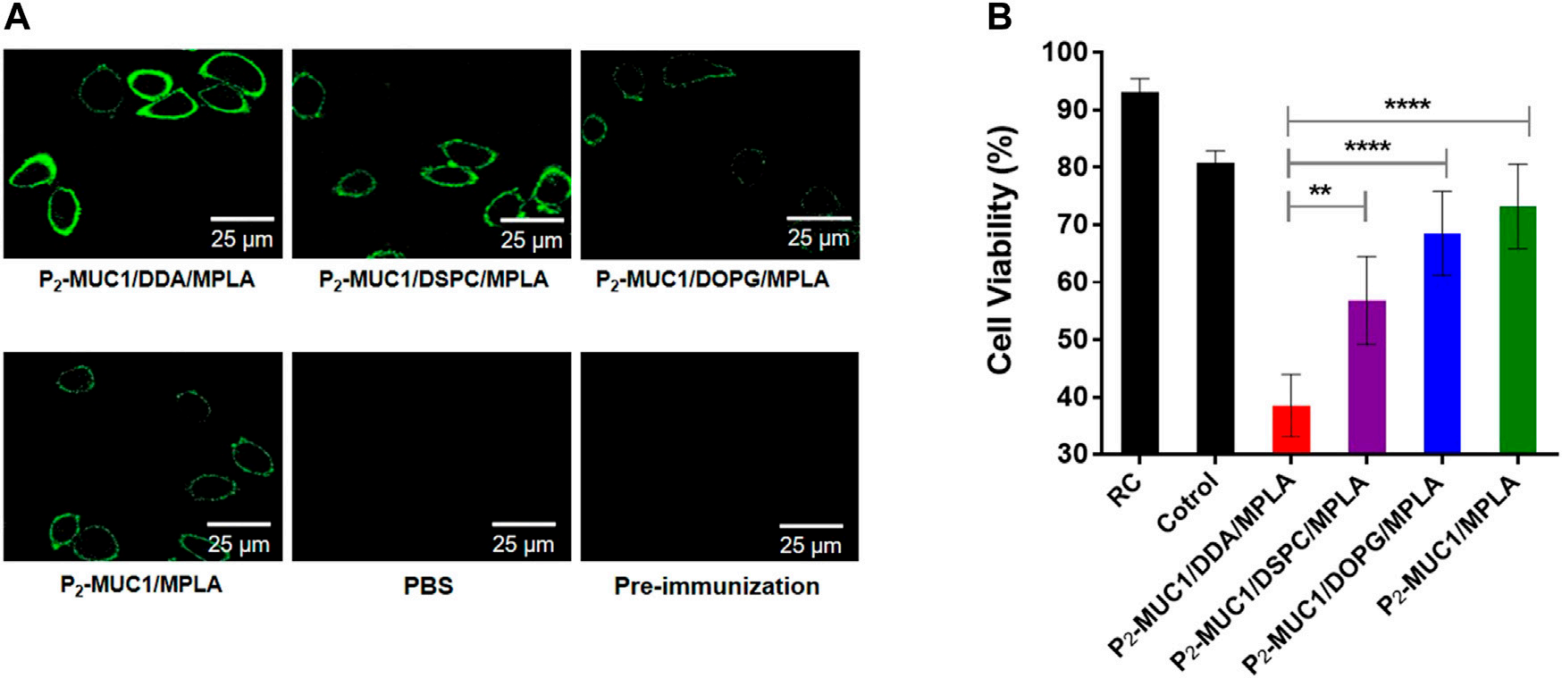
MUC1-specific antibodies recognize MUC1-positive breast cancer cell line (MCF-7). **(A)** Confocal fluorescence microscopy images of the cells stained with pre-immunization sera, sera from PBS and the third immunization antisera collected from P_2_-MUC1/DDA/MPLA, P_2_-MUC1/DSPC/MPLA, P_2_-MUC1/DOPG/MPLA and P_2_-MUC1/MPLA group. (Scale bar = 25 μm). **(B)** MTT test for complement-dependent cytotoxicity. Differences were determined by one-way ANOVA and Tukey’s HSD test (PBS was used as control). Asterisks indicate statistically significant differences (****p < .0001, **p < .01). Data are the mean SD of five mice and are representative of five independent experiments.

Meanwhile, the ability of antisera to recognize human MCF-7 breast cancer cell line was detected by confocal fluorescence microscopy and flow cytometry techniques. As shown in Figure 4A and Supplementary Figure S2, on one hand, P_2_-MUC1/DDA/MPLA vaccine induced higher levels of antibodies that recognized MUC1 positive MCF-7 cancer cells relative to P_2_-MUC1/DSPC/MPLA and P_2_-MUC1/DOPG/MPLA vaccine. On the other hand, P_2_-MUC1/MPLA vaccine without any auxiliary lipids not only generated lower levels of IL-6 cytokines and antibodies, but also showed low binding affinity. This result indicated that the positively charged liposomes with DDA and MPLA were important to enhance the recognition ability of antibodies to cancer cells.

In addition, to evaluate the potential of antisera to activate the complement system, the complement-dependent cytotoxicity (CDC) assay was performed and the percentage of lysed cells was determined by applying a tetrazolium bromide (MTT) assay. As shown in Figure 4B, when P_2_-MUC1 was used as an antigen, the antisera from DDA/MPLA gourp could more effectively activate the complement system and kill MCF-7 cells than DSPC/MPLA and DOPG/MPLA group. These result reflected that the antigen-specific antibodies induced by cationic DDA/MPLA liposomes can effectively activate the complement system. The co-delivery of adjuvant and antigen was guaranteed by stable liposomes. There is a better electrostatic attraction between the positively charged DDA and the negatively charged MPLA agonist, which enhances the stability of the liposomes (Brandt et al., 2000; Korsholm et al., 2010; Wang et al., 2013) and facilitated the uptake of antigen by antigen presenting cells (APCs). On the other hand, positively charged liposomes may be more easily absorbed and swallowed by negatively charged cell membranes. Therefore, the immune responses were enhanced by P_2_-MUC1/DDA/MPLA liposomal vaccine.

**FIGURE 5:**
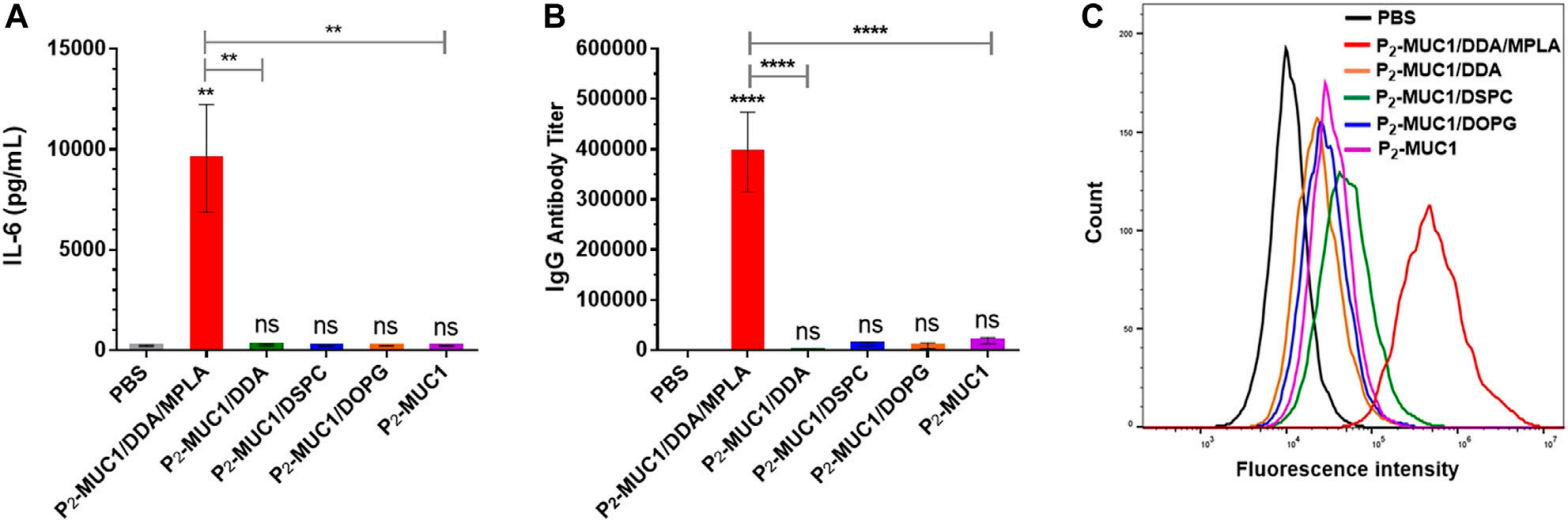
The secretions of cytokines **(A)** and MUC1-specific IgG antibody **(B)** were detected by ELISA (*n* = 5). **(C)** Flow cytometry histograms of the cells stained with sera from PBS and the third immunization antisera collected from P_2_-MUC1/DDA/MPLA, P_2_-MUC1/DDA, P_2_-MUC1/DSPC, P_2_-MUC1/DOPG and P_2_-MUC1 group. Control: phosphate-buffered saline (PBS). ****, *p* < .0001 and **, *p* < .01 compared with the control group by one-way analysis of variance (ANOVA) with the Tukey’s HSD test. Comparisons between different groups were also conducted by ANOVA using Tukey’s HSD test. Data represent the mean ± SD of five mice from three separate experiments.

To determine the role of MPLA adjuvant in vaccines, mice were immunized with P_2_-MUC1/DDA, P_2_-MUC1/DSPC, P_2_-MUC1/DOPG and P_2_-MUC1. As shown in Figure 5, the results showed that only administration with MPLA increased the secretion levels of IL-6 cytokines (Figure 5A) and IgG antibody titers (Figure 5B) in mice. Moreover, the recognition potential of different groups was also assessed (Figure 5C). Only the antisera of mice vaccinated with MPLA showed a strong binding ability to the target cells MCF-7, which indicated that MPLA played an important role to enhance the anti-MUC1 immune responses and improve the recognization ability of antibodies to MCF-7 cells.

### Lipid Modification of MUC1 Glycopeptide Improved Immune Responses

**FIGURE 6:**
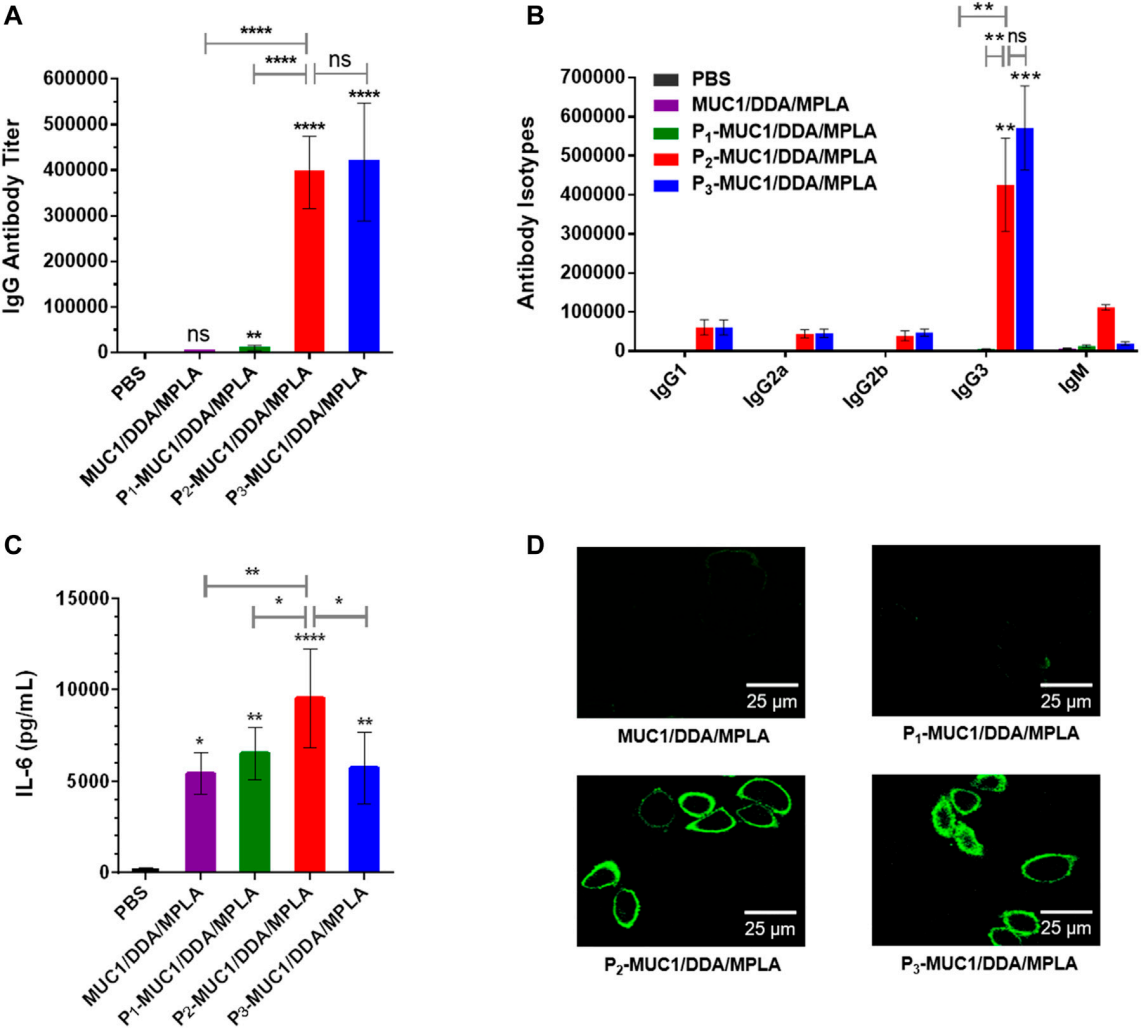
The secretions of MUC1-specific IgG antibody **(A)**, antibody isotypes **(B)** and cytokines **(C)** were detected by ELISA (*n* = 5). **(D)** Confocal fluorescence microscopy images of the cells stained with sera from PBS and the third immunization antisera collected from MUC1/DDA/MPLA, P_1_-MUC1/DDA/MPLA, P_2_-MUC1/DDA/MPLA, P_3_-MUC1/DDA/MPLA. The images are representative of five independent experiments (Scale bar = 25 μm).

To explore the influence of lipid modification, equimolar amounts of MUC1 glycopeptide, P_1_-MUC1, P_2_-MUC1 and P_3_-MUC1 were employed in the DDA/MPLA vaccines design. The results indicated that P_2_-MUC1/DDA/MPLA induced stronger anti-MUC1 specific IgG antibody responses in mice (110 and 38-fold higher IgG titers than MUC1/DDA/MPLA and P_1_-MUC1/DDA/MPLA at day 42, respectively). The IgG antibodies elicited by P_3_-MUC1/DDA/MPLA was comparable to P_2_-MUC1/DDA/MPLA (Figure 6A).

Meanwhile, the sera on day 14 of P_2_-MUC1/DDA/MPLA group also showed high IgG antibody titers, indicating that P_2_-MUC1/DDA/MPLA could rapidly elicit robust immune responses (Supplementary Figure S4). Next, the anti-MUC1 IgG antibody subclasses (IgG1, IgG2a, IgG2b, and IgG3) titers on day 42 were also analyzed (Figure 6B). Except for groups immunized with P_2_-MUC1/DDA/MPLA and P_3_-MUC1/DDA/MPLA, other groups were unable to induce an effective antibody immune response. No significant difference was observed between P_2_-MUC1/DDA/MPLA and P_3_-MUC1/DDA/MPLA in IgG subtypes titers. Interestingly, the level of IgG3 was remarkably higher than that of IgG1, which may be partly attributed to the fact that MPLA is a T helper type 1-like (Th1) adjuvant (Korsholm et al., 2010; Gao and Guo, 2018).

In addition, P_2_-MUC1/DDA/MPLA produced higher levels of IL-6 cytokines compared to MUC1, lipidated P_1_-MUC1 or P_3_-MUC1 with positively charged DDA and MPLA adjuvant (Figure 6C). As shown in Figure 6D, compared with P_3_-MUC1/DDA/MPLA group, P_2_-MUC1/DDA/MPLA group showed a similar fluorescence intensity, which was significantly stronger than other vaccine candidates (MUC1/DDA/MPLA and P_1_-MUC1/DDA/MPLA). This may be due to the fact that lipid modification of MUC1 glycopeptides promoted self-assembly and formation of liposomes, which enhanced immune responses by presenting the multivalent antigens and increasing uptake by APCs. Given that P_2_-MUC1 is easier to synthesize and prepare than P_3_-MUC1 and can be assembled into liposomes with smaller particle sizes (Supplementary Figure S1), dipalmitoyl lipid anchors are the best lipid anchors.

## Conclusion

In conclusion, we developed liposomal vaccines containing auxiliary lipids of different charges, MUC1 lipoglycopeptides and MPLA adjuvant. Compared with the negatively charged DOPG and the neutral DSPC, the positively charged DDA induced stronger antigen-specific immune responses. In addition, we confirmed that the amphiphilic P_2_-MUC1 glycopeptide promoted the assembly of liposomal vaccines and significantly enhanced the recognition ability of antibodies to MCF-7 cells. More importantly, the sera from P_2_-MUC1/DDA/MPLA gourp could effectively activate the complement system and kill MCF-7 cells. The results showed that the strategy of coadministration of lipoglycopeptide and liposomal DDA/MPLA is a convenient platform for building antitumor vaccines.

## Data Availability

The original contributions presented in the study are included in the article/Supplementary Material, further inquiries can be directed to the corresponding authors.
