# Supplementary material for: MUC1 Specific Immune Responses Enhanced by Coadministration of Liposomal DDA/MPLA and Lipoglycopeptide
Source: Front Chem. 2022 Feb 4;10:814880. doi: 10.3389/fchem.2022.814880 (PMC8854779; doi:10.3389/fchem.2022.814880)
Supplement: Supplementary file 1 [file Presentation1.pdf]

Supplementary Material

MUC1 Specific Immune Responses Enhanced by Coadministration of Liposomal DDA/MPLA and Lipoglycopeptide

Jing-Jing Du[1†], Shi-Hao Zhou[2†], Zi-Ru Cheng[1], Wen-Bo Xu[2], Ru-Yan Zhang[2], Long-Sheng Wang[3*], Jun Guo[2*]

This file includes:

General information and synthetic methods

Vaccine formulation and physical characteristics of liposomes

Immunological evaluation

Biological testing methods

HPLC, ESI-MS and MALDI-TOF-MS spectrometry

Supplemental Schemes S1 to S6

Supplemental Table S1

Supplemental Figures S1 to S16

Table of contents

1. General information and synthetic methods....S3

1.1. General information....S3

1.2. General synthetic scheme for MUC1 (1)....S4

1.3. General synthetic scheme for Pam-MUC1 (2)....S5

1.4. General synthetic scheme for Pam_2-MUC1 (3)....S6

1.5. General synthetic scheme for Pam_3-MUC1 (4)....S7

1.6. Synthesis of glycopeptide squaric acid monoamide (5) and BSA-MUC1 (6)....S8

2. Vaccine formulation and physical characteristics of liposomes....S9

2.1. Vaccine formulation and immunization....S9

2.2. Physical characteristics of the liposome formulations....S10

3. Immunological evaluation....S11

3.1. Analysis of the binding of antisera to MCF-7 cell....S11

3.2. Fluorescent microscopy....S12

3.3. The titers of anti-MUC1 antibodies IgG (14, 28, 42 day)....S13

3.3. Preliminary evaluation of the safety of weight change....S13

4. Biological testing methods....S14

5. Supplementary references....S15

6. HPLC, ESI-MS and MALDI-TOF-MS spectrometry....S16

1. General information and synthetic methods

1.1 General information

All L-amino acids and pre-loaded resins were obtained from GL Biochem (Shanghai, China). Anhydrous dichloromethane (DCM) was obtained from the drying solvent system (passed through CaH_2) and can be used without further drying. The purchased anhydrous dimethylformamide (DMF) was stored on the active molecular sieves (4Å). Distearoylglycerophosphocholine (DSPC) was purchased from TCI (Japan) and Energy Chemical (Shanghai, China). Dimethyldioctadecylammonium (DDA) and dioleoylphosphatidylglycerol (DOPG) were purchased from Energy Chemical (Shanghai, China) and Innochem (Beijing, China). Monophosphoryl Lipid A (MPLA) was purchased from Avanti Polar Lipids, Inc. Acetonitrile for HPLC was purchased from Sigma Aldrich (USA). *N*,*N*-Diisopropylethylamine (DIPEA) and trisopropylsilane (TIPS) were purchased from Energy Chemical (Shanghai, China). DCM, DMF, methanol (MeOH), hydrazine hydrate and piperidine were purchased from Sinopharm Chemical Reagent Co (Shanghai, China). Trifluoroacetic acid (TFA) was purchased from Aladdin (Shanghai, China). Palmitic acid (Pam-OH) was purchased from Macklin Biochemical Co (Shanghai, China). Benzotriazol-1-yl-oxytripyrrolidinophosphonium hexafluorophosphate (PyBOP), 2-(7-azabenzotriazol-1-yl)-*N*,*N*,*N'*,*N'*-tetramethyluronium hexafluorophosphate (HATU) and 3H-[1,2,3]-triazolo[4,5-b]pyridin-3-ol (HOAt) were purchased from Bidepharm (Shanghai, China). Semi-preparative HPLC was performed on an Agilent 1260 Series system equipped with a C18 column (Agilent, 250 × 9.2 mm, 5 μm) at a flow rate of 4.0 mL/min using a mobile phase of solvent A (water with 0.1% trifluoroacetic acid) and solvent B (acetonitrile HPLC-grade with 0.1% trifluoroacetic acid). UV absorption signals were detected with an UV detector at wavelength of 200-280 nm. Lipopeptides were separated on a C8 column (Agilent, 250 × 9.2 mm, 5 μm) also at a flow rate of 4 mL/min. Matrix-assisted laser desorption/ionization time of flight (MALDI-TOF) MS was performed on an AB SCIEX 5800 spectrometer. Horseradish peroxidase-conjugated goat anti-mouse IgG, IgM and Alexa Fluor 488-conjugated goat anti-mouse IgG (H+L) were purchased from Jackson ImmunoResearch (USA). Horseradish peroxidase-conjugated goat anti-mouse IgG1, IgG2a, IgG2b and IgG3 were purchased from Southern Biotechnology (USA & Canada). IL-6 mouse ELISA kit was purchased from Biolegend (USA & Canada). Standard rabbit complement was purchased from Cedarlane (Canada). Fluorescence microscopy images were captured on an OLYMPUS IX71. Confocal microscopy images were captured on a Leica TCS SP8. The optical density (OD) of enzyme-linked immunosorbent assay (ELISA) was measured on a BioTek SYNERGY H1. Fluorescence activated cell sorting (FACS) was analyzed on a BD Accuri flow cytometer.

1.2 General synthetic scheme for MUC1 (1)

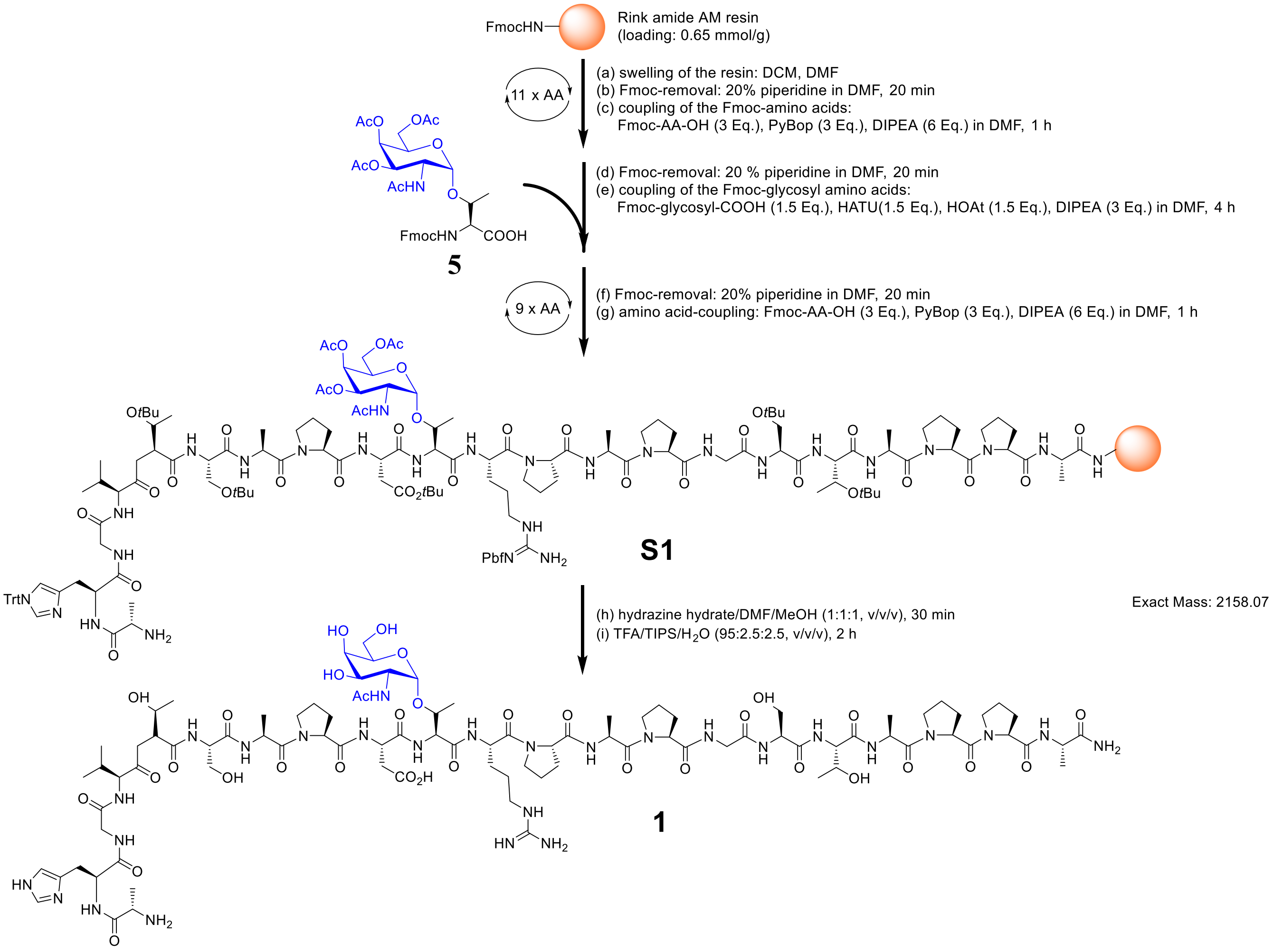

Scheme S1. Preparation of MUC1 **(1)**.

MUC1 (1) was synthesized using the Fmoc-SPPS strategy, starting from Rink amide AM resin. The crude product was dissolved in 30% acetonitrile/water and further purified by semi-preparative HPLC. The compound **1** was determined by analytical HPLC (see Figure S6) (column: Agilent C18, gradient: water/acetonitrile + 0.1% TFA, 0 min (95:5) → 10 min (15:85) → 20 min (95:5), 20 min. Rt = 6. 631min; yield 20 mg (35%). ESI-MS: (see Figure S7): Calcd for $C_{91}H_{146}N_{28}O_{33}$ $[M+2H]^{2+}$ m/z = 1080.03, $[M+2K]^{2+}$ m/z = 1118.03; observed: 1080.81 $[M+2H]^{2+}$; 1118.88 $[M+2K]^{2+}$.

1.3 General synthetic scheme for P1-MUC1 (2)

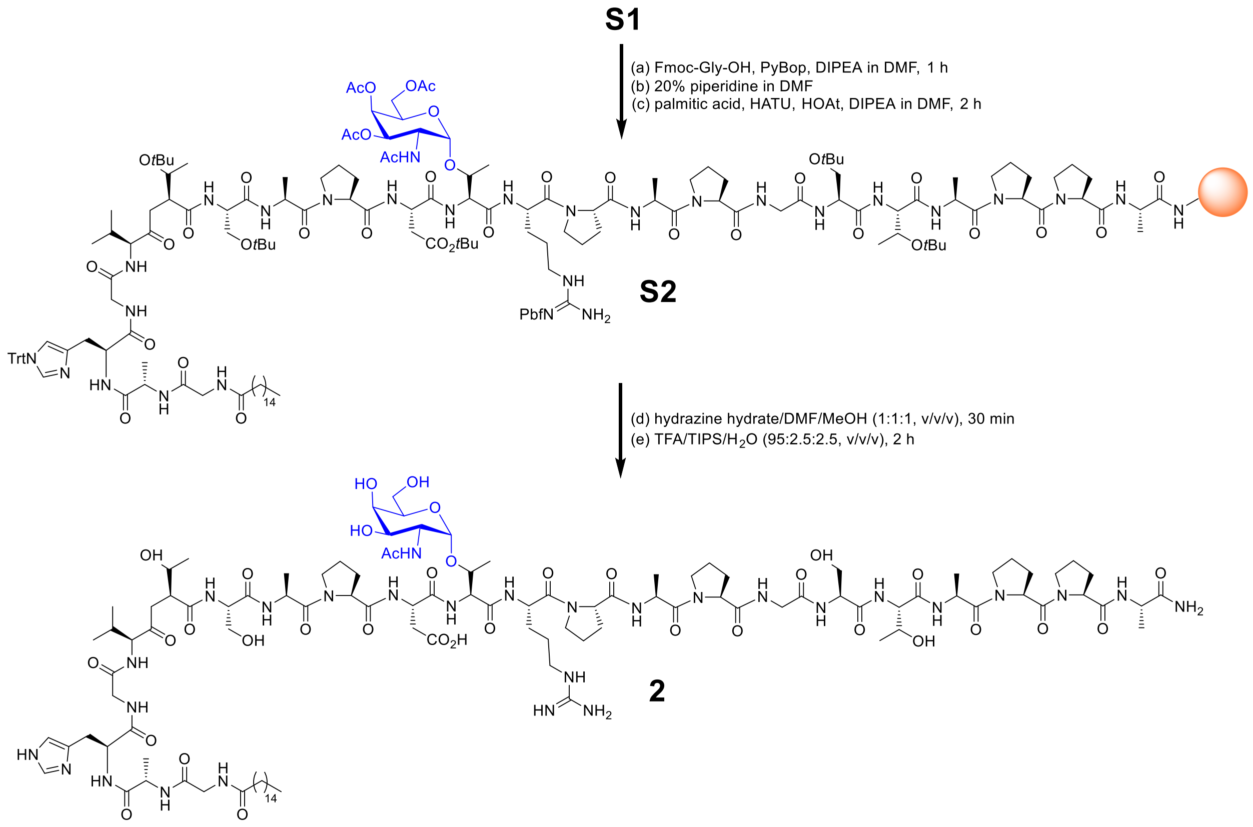

Scheme S2. Preparation of P1-MUC1 (**2**).

P1-MUC1 (2) was synthesized using the Fmoc-SPPS strategy, starting from the resin-bound glycopeptide **S2**. The crude product was dissolved in 50% acetonitrile/water and purified by semi-preparative HPLC. The compound **2** was determined by analytical HPLC (see Figure S8) (column: Agilent C8, gradient: water/acetonitrile + 0.1% TFA, 0 min (65:35) → 15 min (0:100) → 20 min (65:35), 20 min. Rt = 9.749 min; yield 32 mg (26%); ESI-MS: (see Figure S9): Calcd for $C_{109}H_{179}N_{29}O_{35}$ $[M+2H]^{2+}$ m/z = 1228.66, observed: 1228.64 $[M+2H]^{2+}$.

1.4 General synthetic scheme for P2-MUC1 (3)

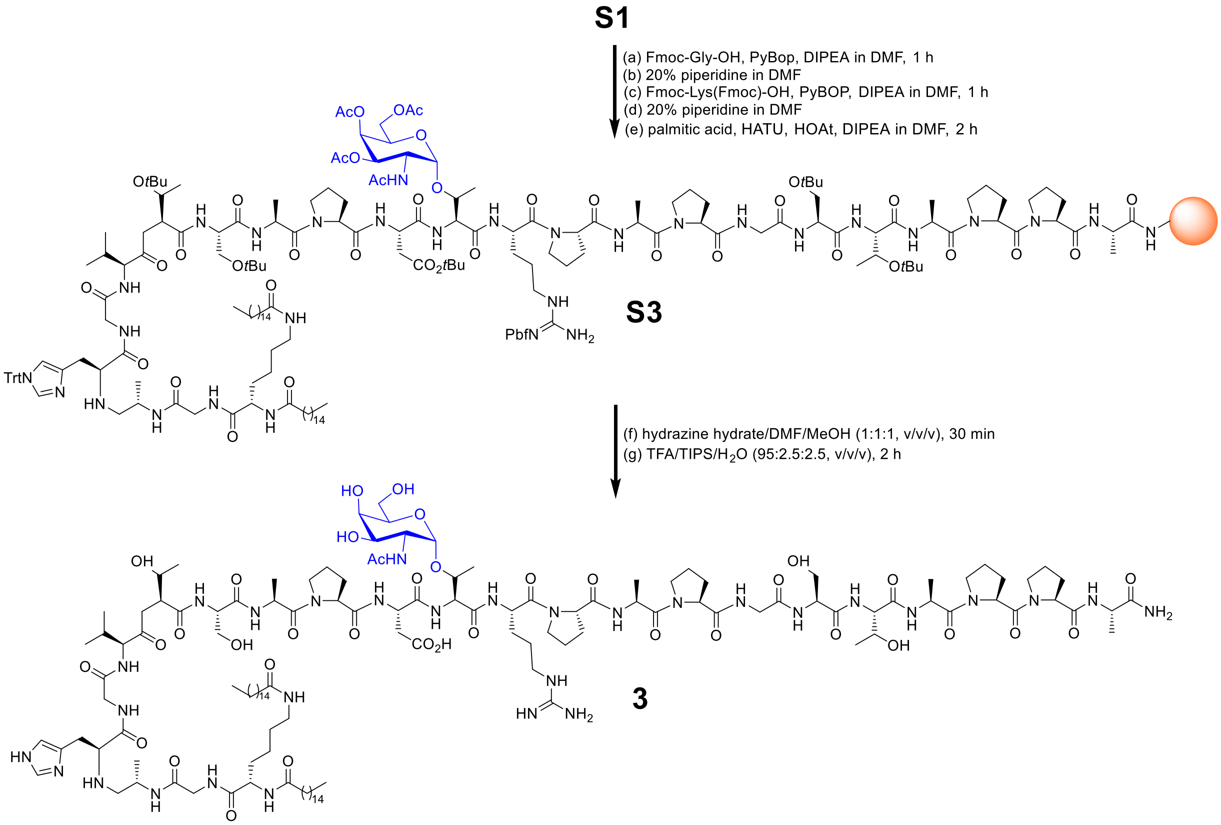

Scheme S3. Preparation of P2-MUC1 (**3**).

P2-MUC1 (3) was synthesized using the Fmoc-SPPS strategy, starting from the resin-bound glycopeptide **S3**. The crude product was dissolved in 50% acetonitrile/water and purified by semi-preparative HPLC. The compound **3** was determined by analytical HPLC (see Figure S10) (column: Agilent C8, gradient: water/acetonitrile + 0.1% TFA, 0 min (65:35) → 15 min (0:100) → 20 min (65:35), 20 min. R_t = 10.677 min; yield 19 mg (15%); ESI-MS: (see Figure S11): Calcd for $C_{131}H_{221}N_{31}O_{37}$ $[M+2H]^{2+}$ *m/z* = 1411.82; observed: 1412.92 $[M+2H]^{2+}$.

1.5 General synthetic scheme for P3-MUC1 (4)

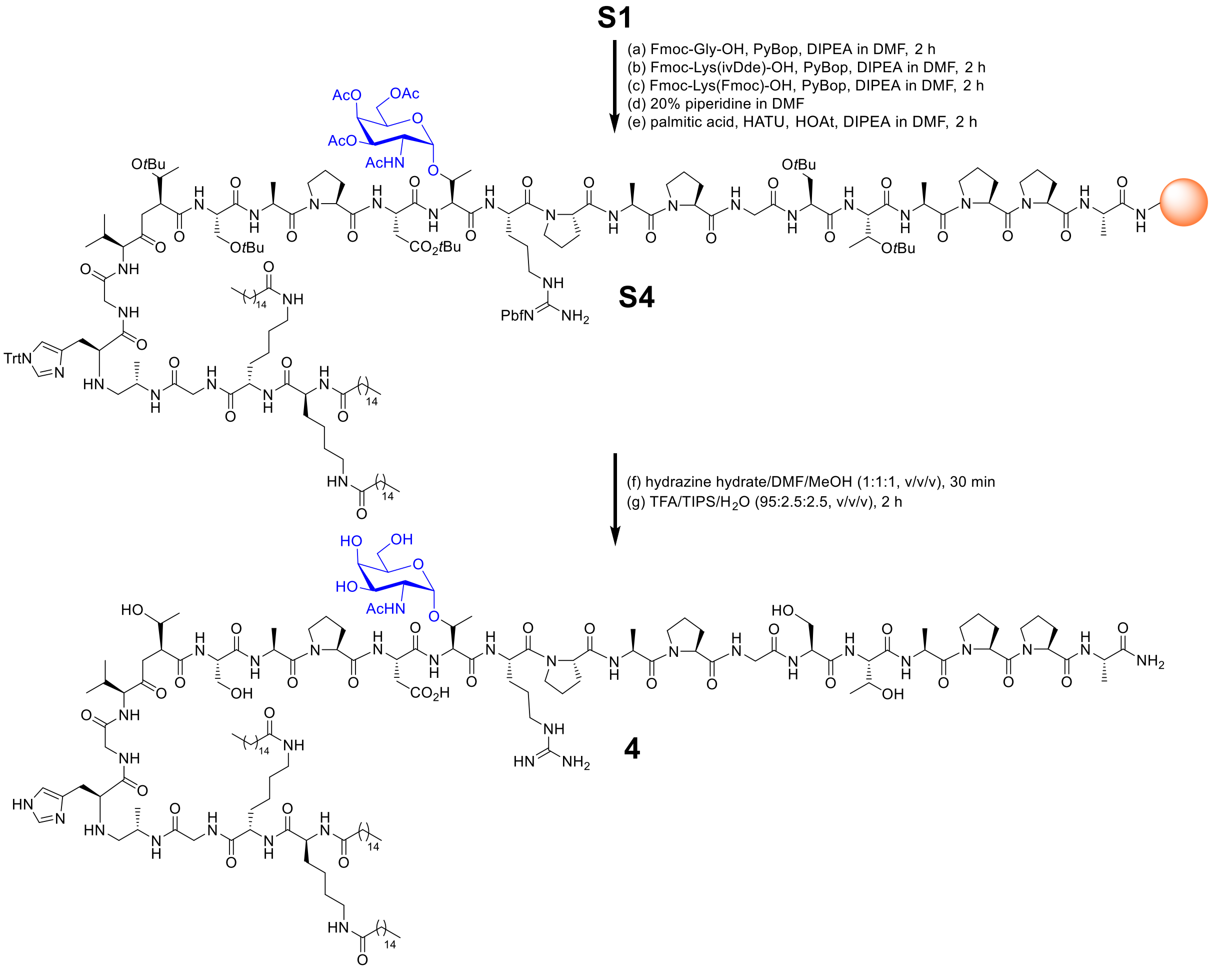

Scheme S4. Preparation of P3-MUC1 (**4**).

P3-MUC1 (4) was synthesized using the Fmoc-SPPS strategy, starting from Rink amide AM resin. The crude product was dissolved in 50% acetonitrile/water and further purified by semi-preparative HPLC. The compound **4** was determined by analytical HPLC (see Figure S12) (column: Agilent C8, gradient: water/acetonitrile + 0.1% TFA, 0 min (20:80) → 15 min (0:100) → 20 min (0:100), 15 min. R_t = 13.531 min; yield 16 mg (10%); ESI-MS: (see Figure S13): Calcd for $C_{153}H_{263}N_{33}O_{39}$ $[M+2H]^{2+}$ *m/z* = 1594.98, observed: 1595.57 $[M+2H]^{2+}$.

1.6 General synthetic schemes for MUC1 glycopeptide squaric acid monoamide (5) and BSA-MUC1 (6)

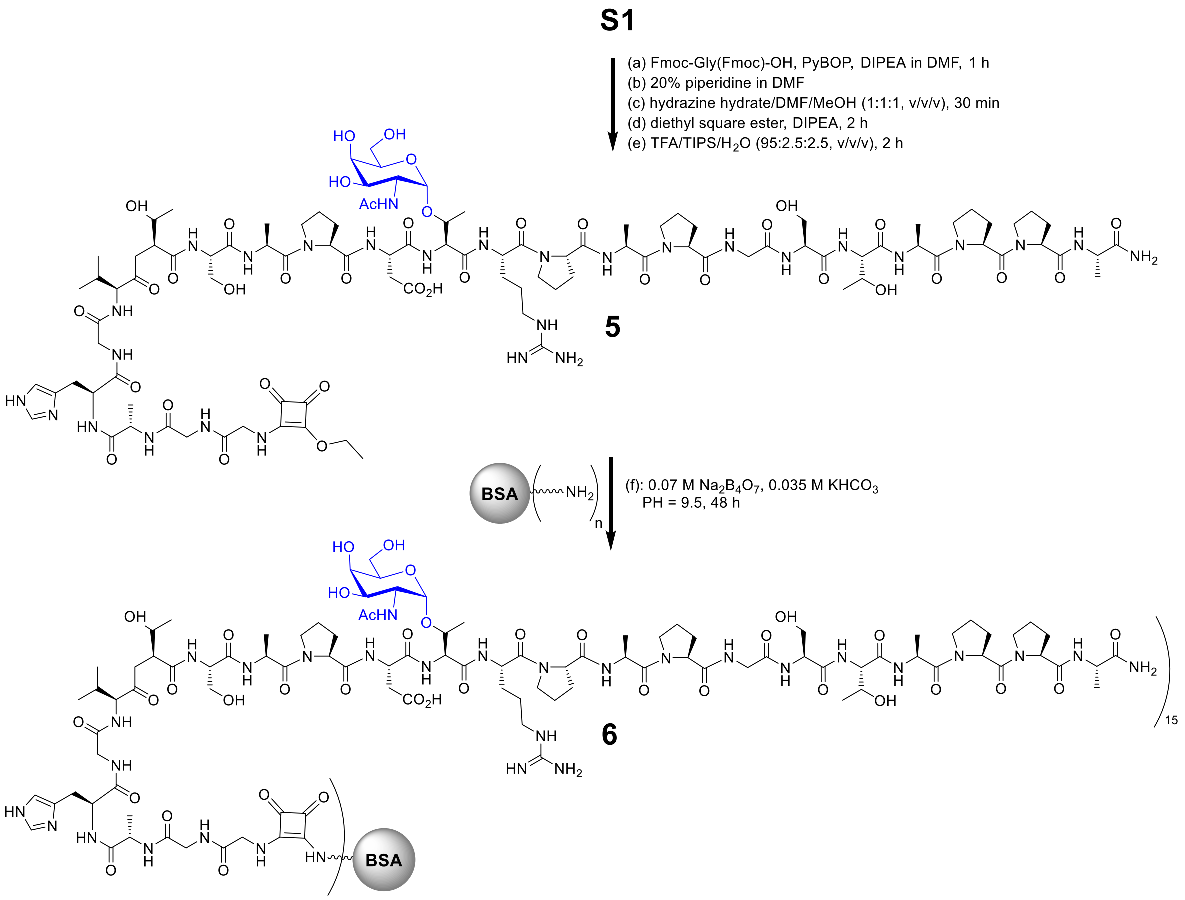

Scheme S5. Preparation of BSA-MUC1 (**6**).

Glycopeptide squaric acid monoamide (5) was synthesized using the Fmoc-SPPS strategy, starting from the resin-bound glycopeptide **S5**. The compound **5** was dissolved in 30% acetonitrile/water and purified by semi-preparative HPLC. The compound **5** was determined by analytical HPLC (see Figure S14) (column: Agilent C18, gradient: water/acetonitrile + 0.1% TFA, 0 min (95:5) → 12 min (30:70) → 15 min (0:100), 15 min. R_t = 7.782 min; yield 27 mg (40%); ESI-MS: (see Figure S15): Calcd for $C_{101}H_{158}N_{30}O_{38}$ $[M+2H]^{2+}$ *m/z* = 1200.06; observed: 1199.07 $[M+2H]^{2+}$.

BSA-MUC1 (6). Linking the resulting squarate monoamide (**5**) (3.0 µmol, 50 Eq.) to bovine serum albumin (BSA) (4.0 mg, 0.06 µmol) were then achieved in 0.07 M $Na_2B_4O_7$/0.035 M $KHCO_3$ buffer solution at pH 9.5,[3] providing the desired conjugate **6** after dialysis. The antigen loading level of **6** was estimated by MALDI-TOF mass spectrometry and indicated on average fifteen molecules of glycopeptide per molecule of BSA (see Figure S16). The BSA-MUC1 (**6**) was used as coating reagent for ELISA.

2. Vaccine formulation and physical characteristics of liposomes

2.1 Vaccine formulation and immunization

The composition of each vaccine candidate is shown in Table S1. Vaccines **A-D**: the liposomes of mixture of adjuvant (each mouse MPLA 17 μg), antigen (MUC1 for **A**, P_1-MUC1 for **B**, P_2-MUC1 for **C**, and P_3-MUC1 for **D**), and auxiliary lipid DDA in a molar ratio of 10:10:80 were dissolved in a mixture of DCM/MeOH (1:1, v/v, 2 mL); Vaccines **E-G**: the liposomes of mixture of adjuvant (MPLA 17 μg), antigen (P_2-MUC1 28 μg), and auxiliary lipid (DSPC for **E**, DOPG for **F**, no auxiliary lipid for **G)** in a molar ratio of 10:10:80 were dissolved in a mixture of DCM/MeOH (1:1, v/v, 2 mL); Vaccines **H-K**: the liposomes of mixture of antigen (P_2-MUC1 28 μg) and auxiliary lipid (DDA for **H**, DSPC for **I**, DOPG for **J**, no auxiliary lipid for **K)** in a molar ratio of 10:80 were dissolved in a mixture of DCM/MeOH (1:1, v/v, 2 mL), and the solvents were removed under reduced pressure through rotary evaporation, which generated a thin lipid film on the flask wall. This film was hydrated and subjected to freeze/thaw cycles to produce multilamellar vesicles, then the Tris buffer (20 mM, pH 7.4) was added and shaking the mixture under an argon atmosphere at 25 °C for 1 h. The milky suspension was finally sonicated for 20 min to obtain the optimal liposomes.

Vaccines	Auxiliary lipid (80 nmol)	Adjuvant (10 nmol)	Antigen (10 nmol)
**A**	DDA (50 μg)	MPLA (17 ug)	MUC1 (22 μg)
**B**	DDA (50 μg)	MPLA (17 ug)	P_1-MUC1 (25 μg)
**C**	DDA (50 μg)	MPLA (17 ug)	P_2-MUC1 (28 μg)
**D**	DDA (50 μg)	MPLA (17 ug)	P_3-MUC1 (32 μg)
**E**	DSPC (63 μg)	MPLA (17 ug)	P_2-MUC1 (28 μg)
**F**	DOPG (62 μg)	MPLA (17 ug)	P_2-MUC1 (28 μg)
**G**	\	MPLA (17 ug)	P_2-MUC1 (28 μg)
**H**	DDA (50 μg)	\	P_2-MUC1 (28 μg)
**I**	DSPC (63 μg)	\	P_2-MUC1 (28 μg)
**J**	DOPG (62 μg)	\	P_2-MUC1 (28 μg)
**K**	\	\	P_2-MUC1 (28 μg)

Table S1. The composition of each vaccine candidate. The amounts of each component in the table are used for one injection per mouse.

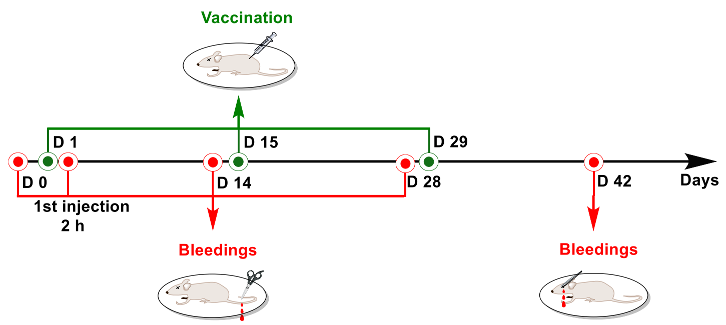

Scheme S6. Study design for MUC1 vaccine candidates in mice[2]. Mice were immunized on the 1st, 15th, and 29th days, and the blood was taken on day 0 before initial immunization, and 14th, 28th, and 42nd days before the boost immunizations.

2.2 Physical characteristics of the liposome formulations

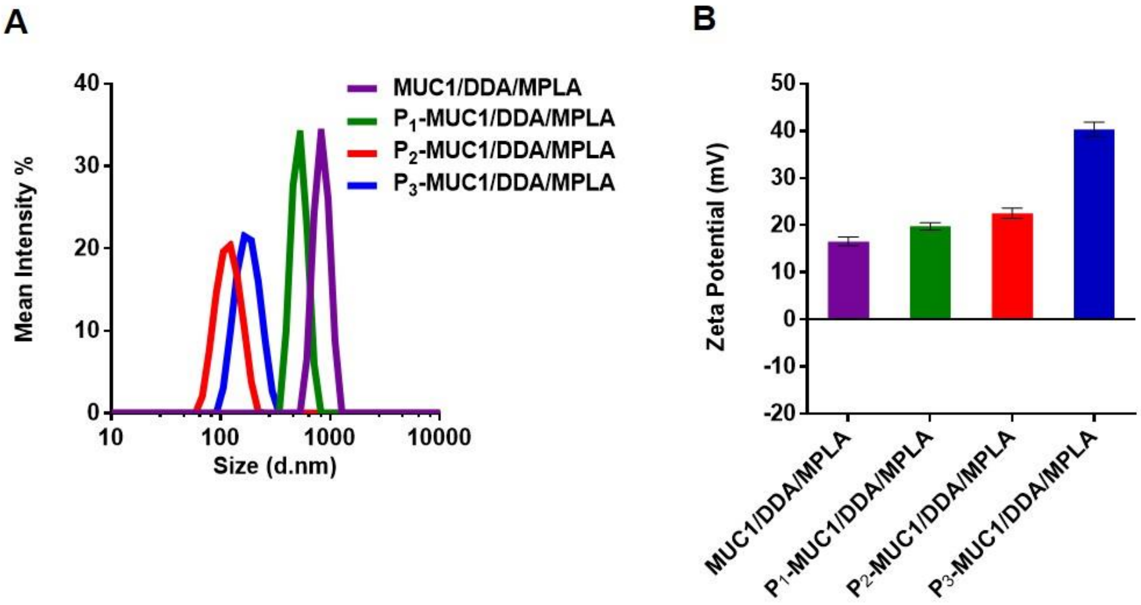

Figure S1. (A) DLS results of vaccine candidates. (B) Zeta potential results of vaccine candidates, values are presented as the mean ± SD (n=3).

3. Immunological evaluation

3.1 Analysis of the binding of antisera to MCF-7 cells

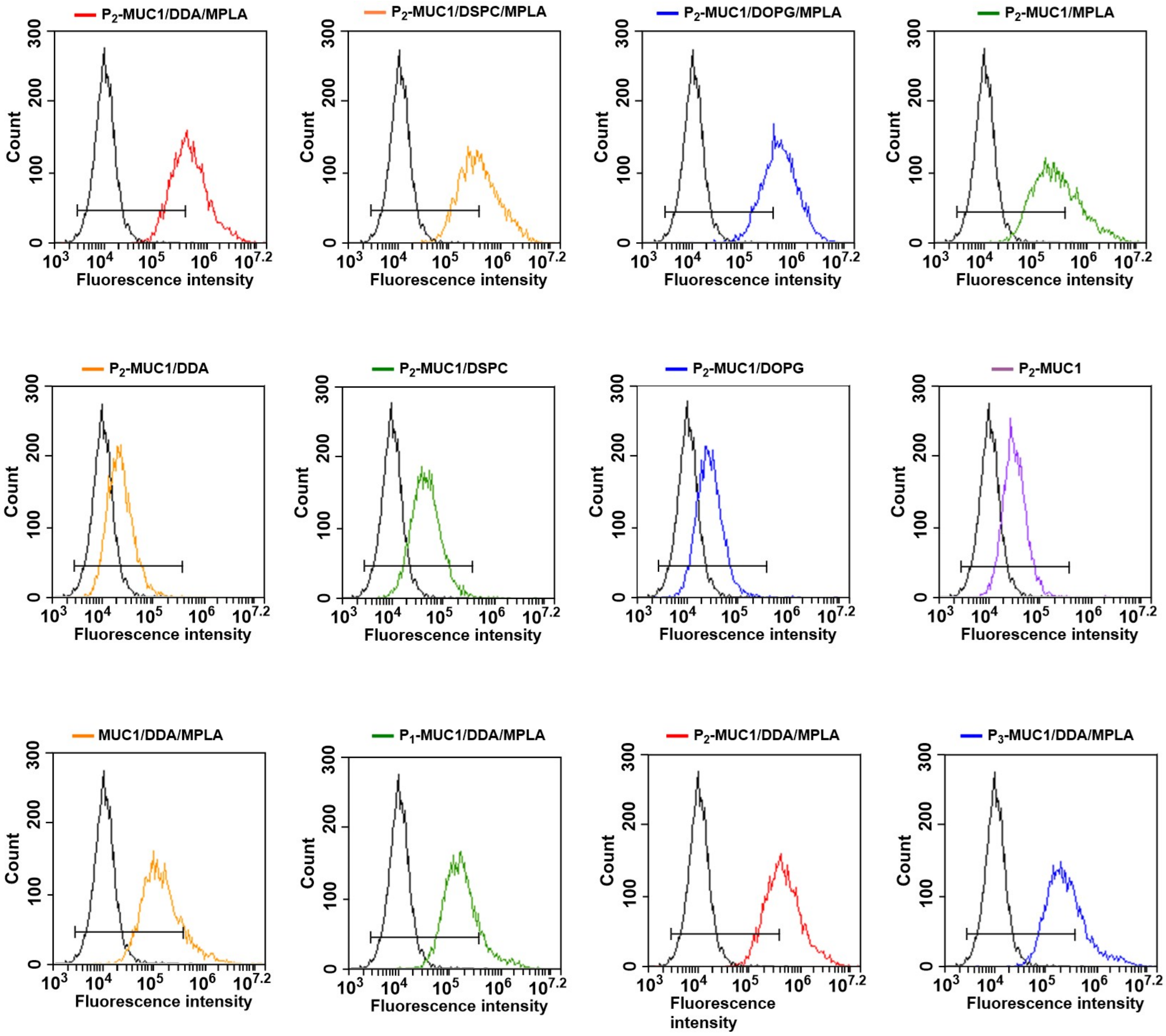

Figure S2. Flow cytometry was used to analyze the binding of antisera induced by all vaccines to MCF-7 tumor cells. Cells incubated with pre-immune sera (black) served as control.

3.2 Fluorescent microscopy

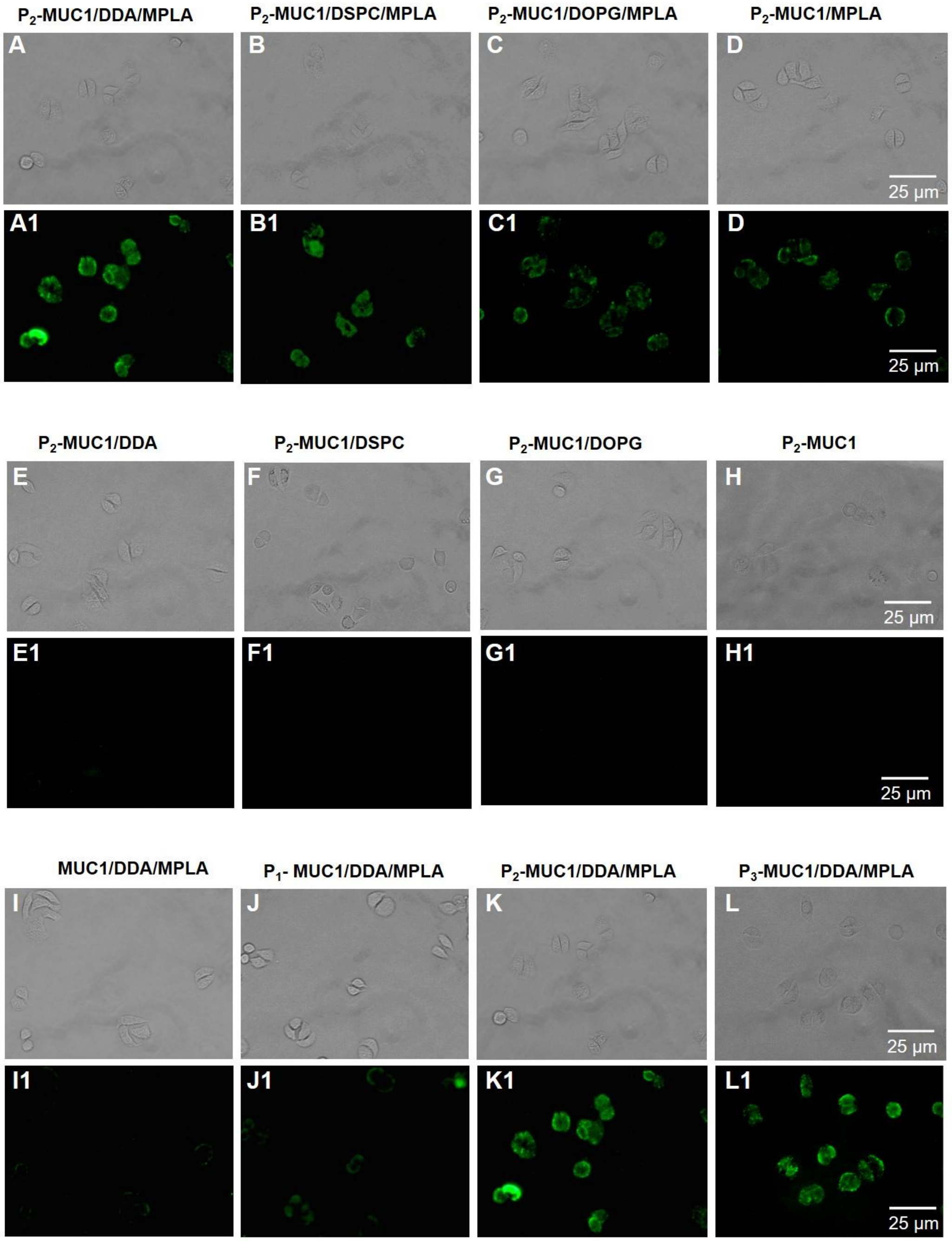

Figure S3. Fluorescence-microscopy staining of breast cancer cells (MCF-7). Top row A-L: bright field images. Bottom row A1-L1: fluorescent images. A-L and A1-L1: the cells were incubated with sera of A-F for 1 h at 4 °C.

3.3 The titers of anti-MUC1 antibodies IgG (14, 28, 42 day)

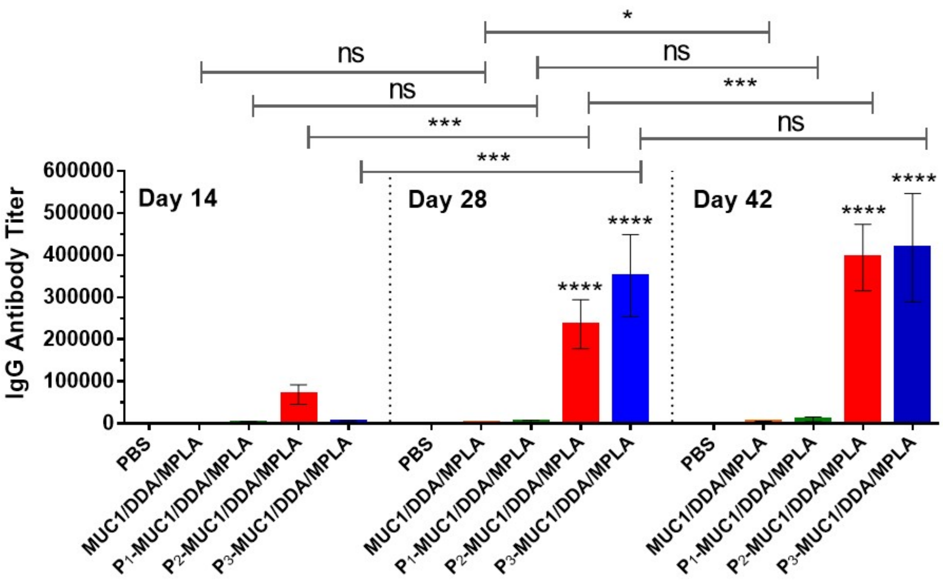

Figure S4. The titers of anti-MUC1 antibodies IgG (14, 28, 42 day) elicited by the vaccine candidates. The data are indicated as the average of two independent experiments ± SEM. *, $P < 0.05$; ***, $P < 0.001$; ****, $P < 0.0001$. ns, no significant difference.

3.4 Preliminary evaluation of the safety of weight change

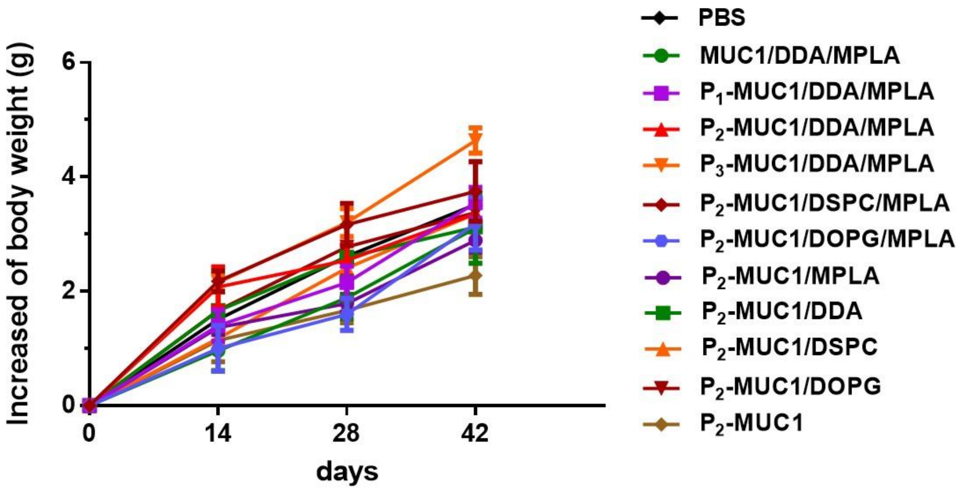

Figure S5. Assessment of the safety of MUC1 vaccines by monitoring the weight change.

4. Biological testing methods

Mouse immunization schedule

BALB/c mice (five per group) were vaccinated via intraperitoneal injection of vaccines (**A-I**) on days 1, 15 and 29 of a 2-week cycle by intraperitoneal injection, sera were collected on 2 h, 24 h and days 14, 28, 42 after the first immunization. All the animal experiments were conducted strictly in accordance with the principles of welfare and ethics of laboratory animals.

Protocol for ELISA

The antibody titers and antibody isotypes generated by the vaccine candidates were measured by ELISA. The biotinylated MUC1 (0.125 μg/mL) and avidin (1.16 μg/mL) were directly dissolved in the prepared $NaHCO_3/Na_2CO_3$ buffer (50 mM, pH 9.5) with a final concentration of 0.125 μg/mL and 1.16 μg/mL, then 96-well ELISA plates (costar 3590) were coated with avidin-biotin-MUC1 complex solution and incubated at 4°C overnight. Then the plates were washed three times with 0.05% Tween PBS and blocked with PBS containing 3% BSA for 1 hour at 37 ℃. After the plates were washed three times with 0.05% PBST, the diluted antisera were added to each well (100 μL/well) and incubated for 1 h at 37 ℃. After washing, the horseradish peroxidase (HRP)-conjugated goat anti-mouse IgG (γ-chain specific), IgM (μ-chain specific), IgG1, IgG2a, IgG2b and IgG3 (1:2000 dilution) in 0.1% BSA/PBS were added to each well (100 µL/well) respectively, and incubated for 1 h at 37 ℃. After washing, the freshly prepared substrate solutions [9.5 mL critic buffer at pH 5.0, 0.5 mL 1.6 mg/mL tetramathyl benzidine (TMB), and 32 µL 3% (w/v) urea hydrogen peroxide] were added to each well (100 µL/well) in the dark for 5 minutes, then 2 M H_2SO_4 (50 µL/well) was added to quench the reaction. Optical density was then measured at a wavelength of 450 nm with a microplate reader (Synergy H1). The titer of antibody was defined as the highest dilution, indicating that the absorbance was 0.1, minus the background. Data were analyzed with the software GraphPad (San Diego, CA).

Cell culture

Human breast tumor cells (MCF-7) were obtained from ATCC (HTB 22) (Rockville, Maryland, USA) and grown in Dulbecco's modified Eagle's medium (DMEM) + 1% penicillin/streptomycin + 10% fetal bovine serum (FBS, Invitrogen). Cell dissociation during passaging was carried out in 0.05% trypsin/EDTA solution (Gibco, Life Technologies, Carlsbad, USA). All cells were maintained at 37°C in humidified atmosphere with 5% CO_2.

Analysis of the binding of antisera to human breast cancer cells

MCF-7 cells were incubated with sera of vaccines **A-I** (diluted 1:50) in fluorescence-activated cell sorting (FACS) buffer (contained PBS (1×), 10% FBS and 0.1% sodium azide) for 1 h at 4 °C. Then cells were washed three times with 1% BSA/PBS and incubated with secondary antibodies (Alexa Fluor 488-conjugated AffiniPure Goat Anti-Mouse IgG (H+L), Jackson ImmunoResearch, diluted 1:50) for 30 min at 4°C. After washing three times with 1% BSA/PBS, the cells were added the FACS buffer and analyzed by flow cytometry

Fluorescent microscopy

MCF-7 cells were grown to 80% confluence and seeded overnight with Dulbecco's modified Eagle's medium (DMEM). After washing with 1% BSA/PBS buffer, the cells were incubated with sera (diluted 1:50) of all vaccines for 1 h at 4 °C. Then culture medium was removed and cells were incubated with secondary antibodies (Alexa Fluor 488-conjugated AffiniPure Goat Anti-Mouse IgG

(H+L), Jackson ImmunoResearch, diluted 1:50) for 30 min at 4 °C. The cell images were obtained with laser scanning by confocal microscope (Leica TCS SP8) and fluorescence microscope (OLYMPUS IX71), respectively.

Cell cytotoxicity

To assess whether the induced antibodies of the antisera from vaccinated mice can mediate complement lysis via activation of the complement dependent cytotoxicity (CDC). For this purpose, MCF-7 cells were incubated with these antisera (at a antisera dilution of 1:50) (50 μL per well) from vaccinated mice, after washing with PBS solution, the prepared rabbit sera (at a rabbit sera dilution of 1:50) in 1% BSA/PBS were added as complement supplier (RC: rabbit complement; RC-inactive: inactivated rabbit complement). MCF-7 cells were incubated with antisera diluted to 1:50 in 1% BSA/PBS (50 μL per well). After washing, the rabbit sera diluted to 1:5 in 1% BSA/PBS, and 50 μL/well of the solution were added. After incubation for 4 hours, 0.5% MTT solution in PBS (MTT: 3-(4,5-dimethyl-2-thiazolyl)-2,5-diphenyl-2-H-tetrazolium bromide) was added (20 μl/well) and incubated for 2 h. After removing the medium, DMSO was added (150 μL/well) and the absorption was analyzed at the wavelength of 490 nm. The survival rate of cells was measured with the following formula. All assays were conducted in five independent experiments.

5. Supplementary references

[1] Yin, X.-G.; Chen, X.-Z.; Sun, W.-M.; Geng, X.-S.; Zhang, X.-K.; Wang, J.; Ji, P.-P.; Zhou, Z.-Y.; Baek, D. J.; Yang, G.-F.; Liu, Z.; Guo, J. IgG Antibody Response Elicited by a Fully Synthetic Two-Component Carbohydrate-Based Cancer Vaccine Candidate with α-Galactosylceramide as Built-in Adjuvant. Org. Lett. 2017, 19, 456–459.

[2] Niu, Y.; Wang, N.; Cao, X.; Ye, X.-S. Efficient Formation and Cleavage of Benzylidene Acetals by Sodium Hydrogen Sulfate Supported on Silica Gel. Synlett 2007, 2007, 2116–2120.

[3] (a) Heidelberg, T.; Martin, O. R. Synthesis of the Glycopeptidolipid of Mycobacterium avium Serovar 4: First Example of a Fully Synthetic C-Mycoside GPL. J. Org. Chem. 2004, 69, 2290–2301. (b) Wipf, P.; Pierce, J. G. Expedient Synthesis of the α-C-Glycoside Analogue of the Immunostimulant Galactosylceramide (KRN7000). Org. Lett. 2006, 8, 3375–3378.

6. HPLC, ESI-MS and MALDI-TOF-MS spectrometry

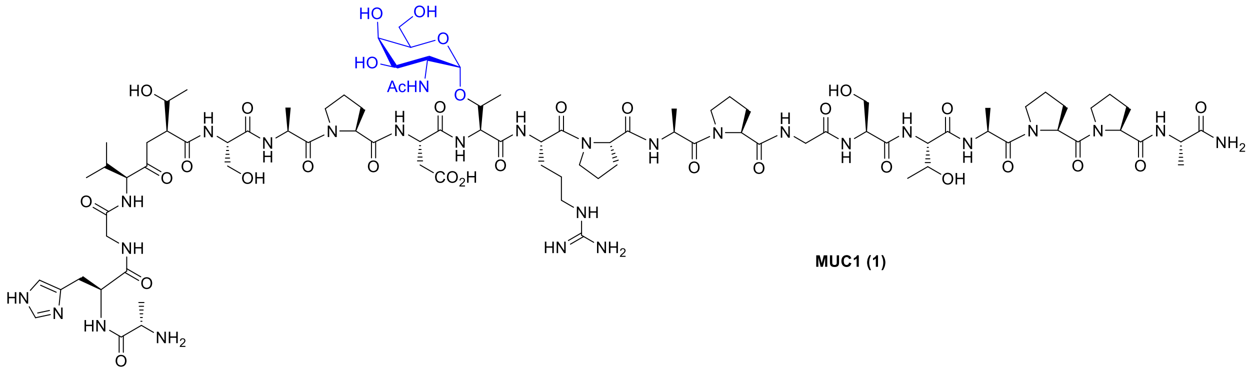

MUC1 (1)

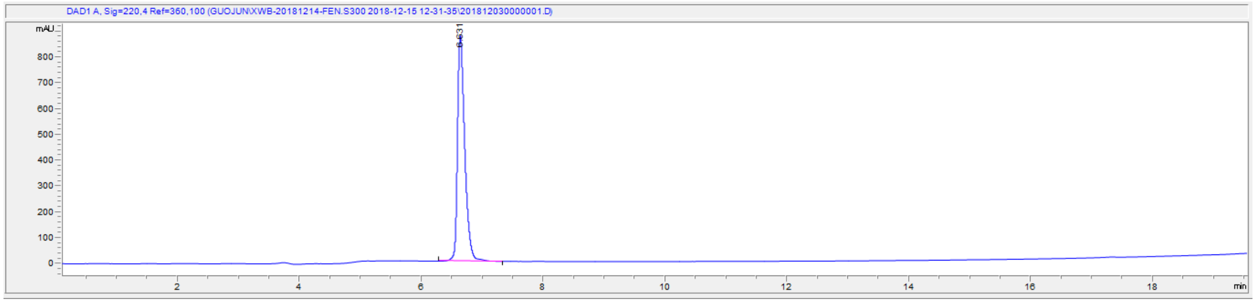

Figure S6. HPLC chromatogram of compound **1**.

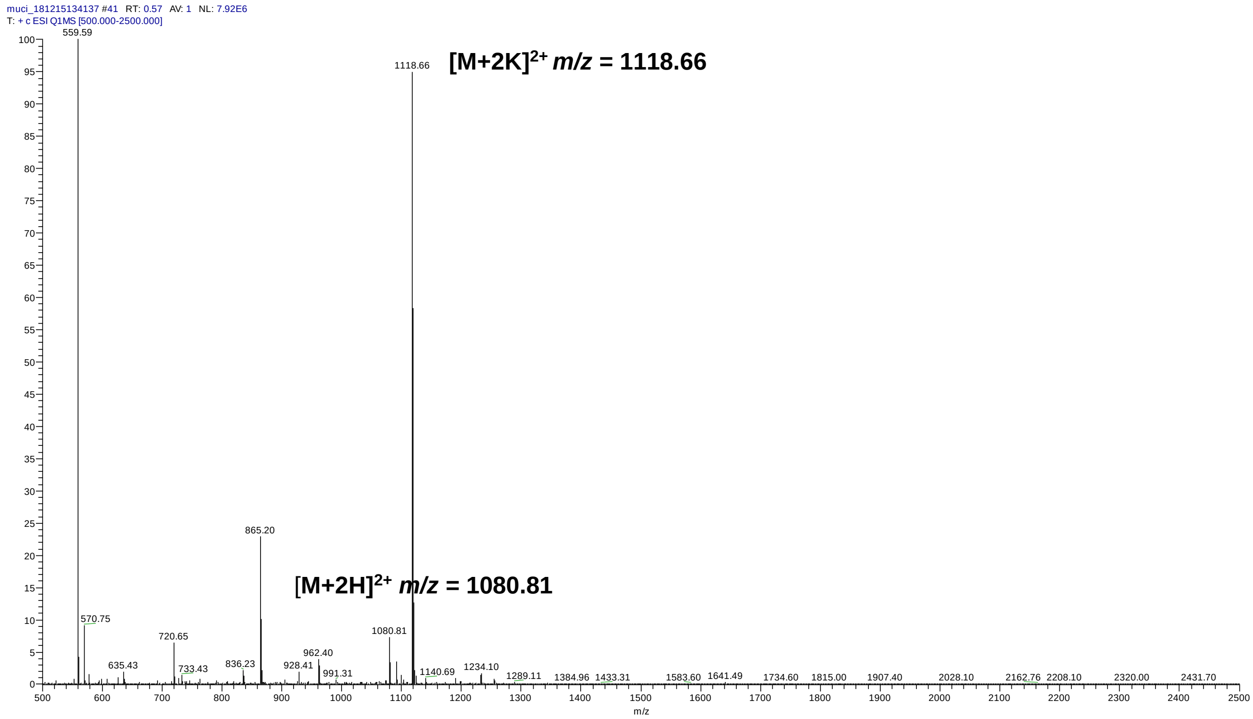

Figure S7. ESI-MS data of compound **1**.

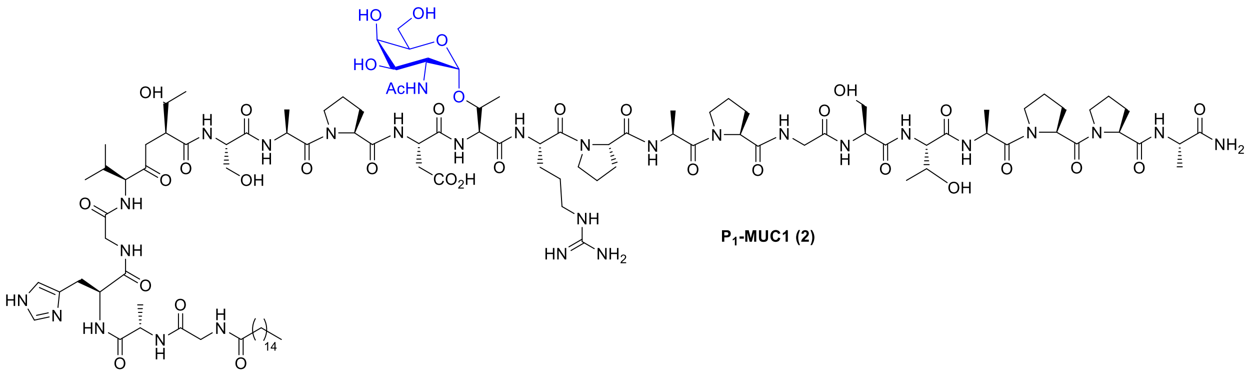

P_1-MUC1 (2)

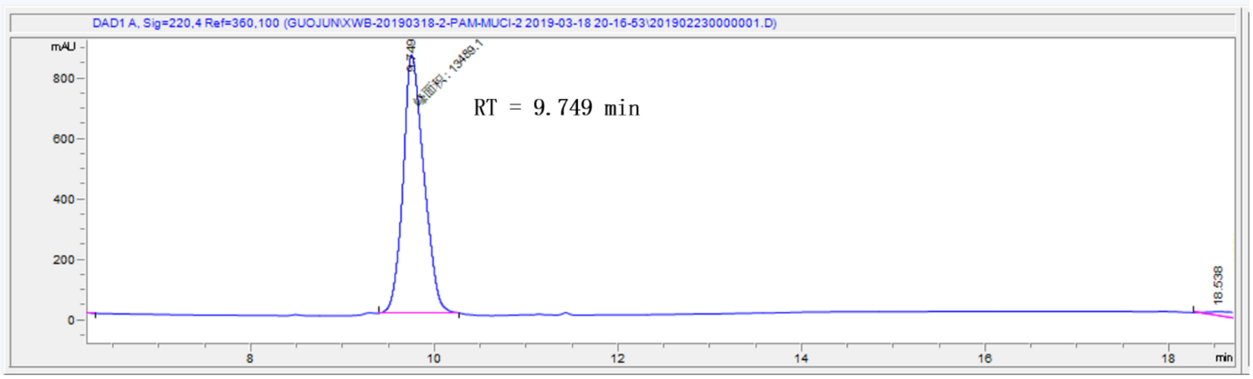

Figure S8. HPLC chromatogram of compound **2**.

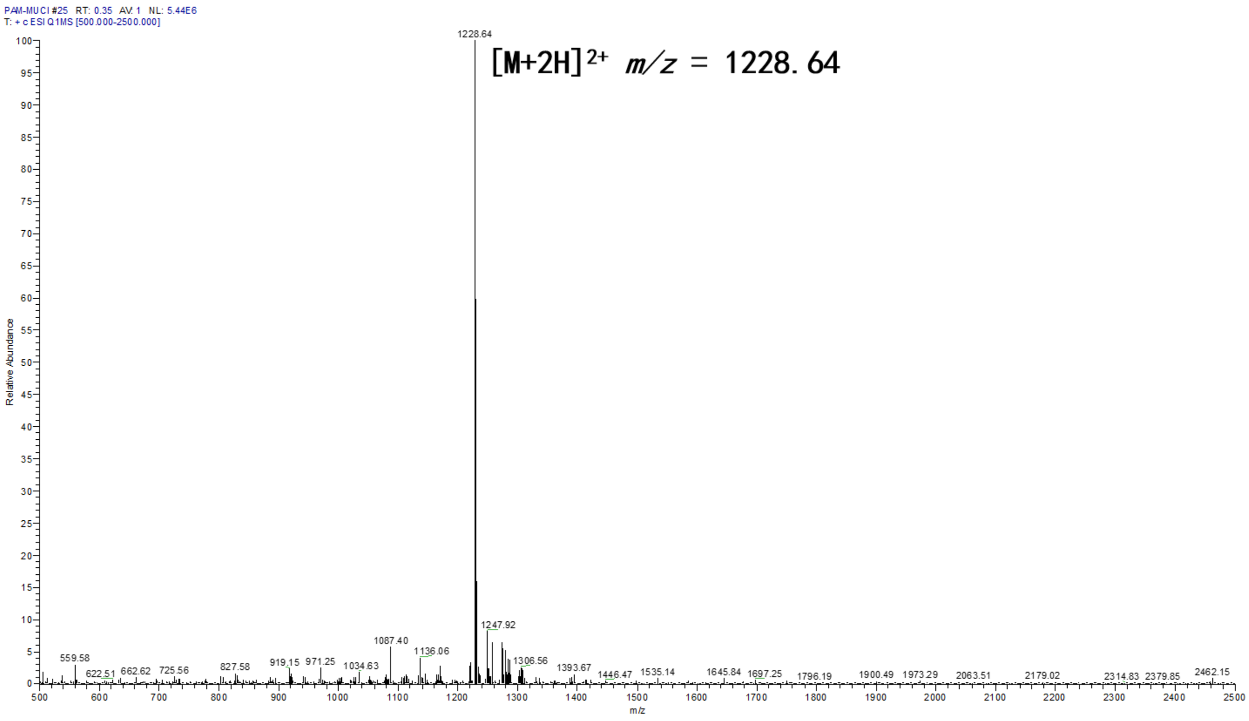

Figure S9. ESI-MS data of compound **2**.

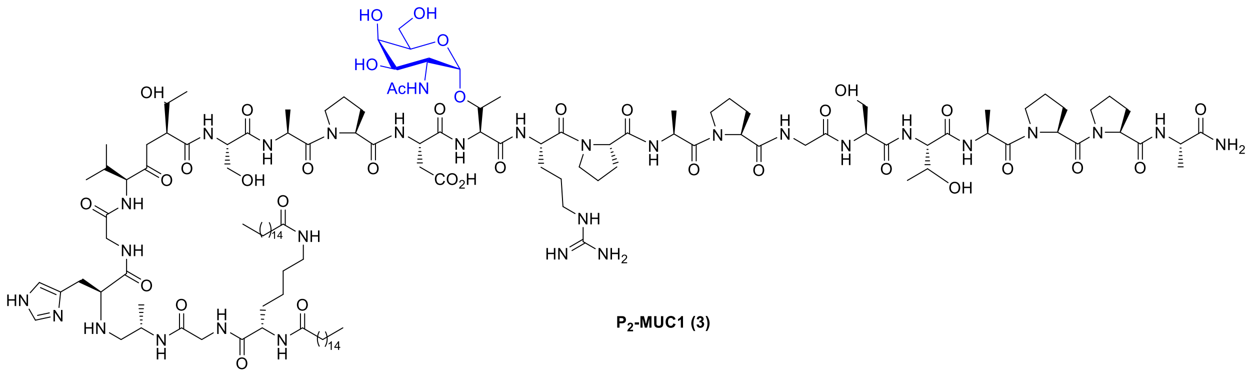

P_2-MUC1 (3)

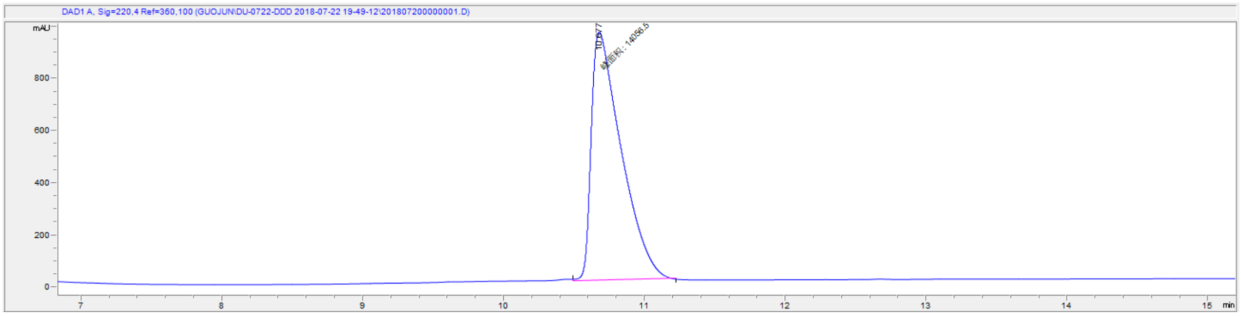

Figure S10. HPLC chromatogram of compound **3**.

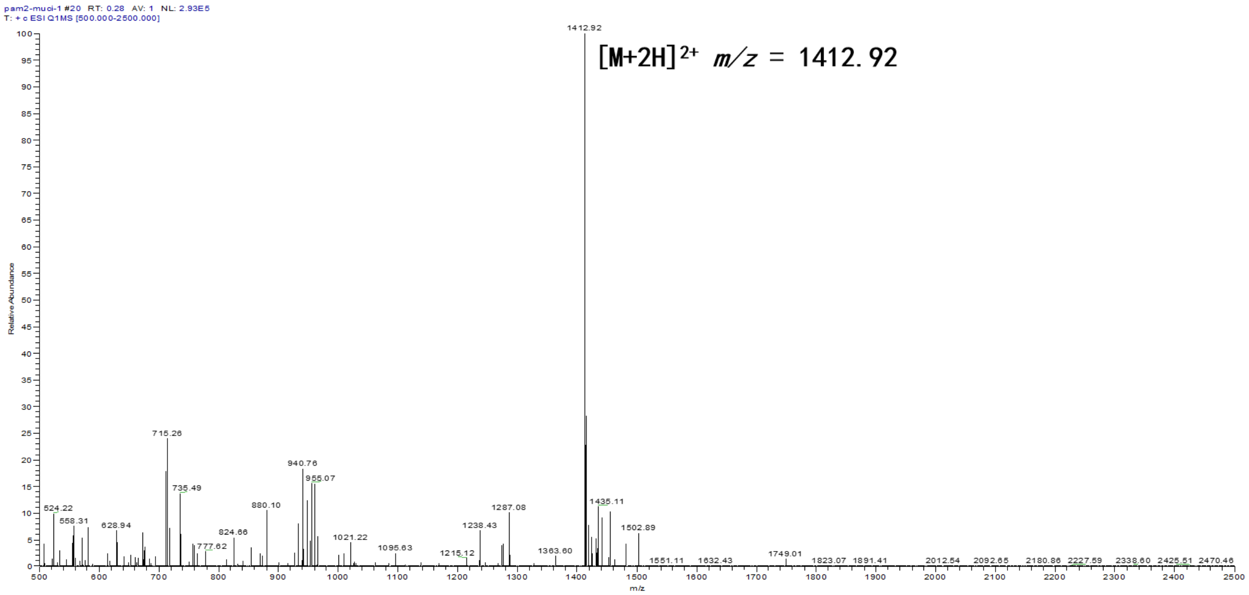

Figure S11. ESI-MS spectra of compound **3**.

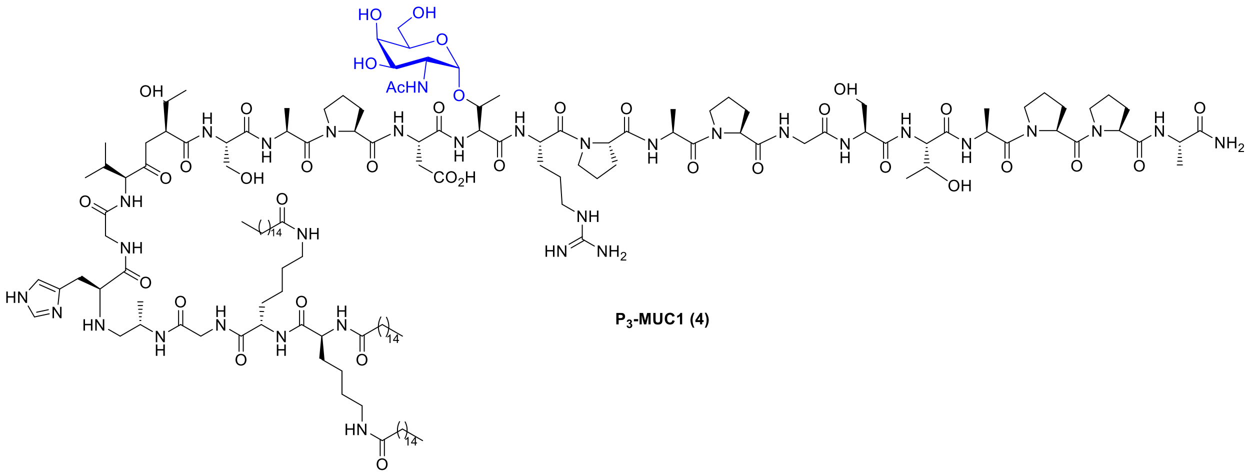

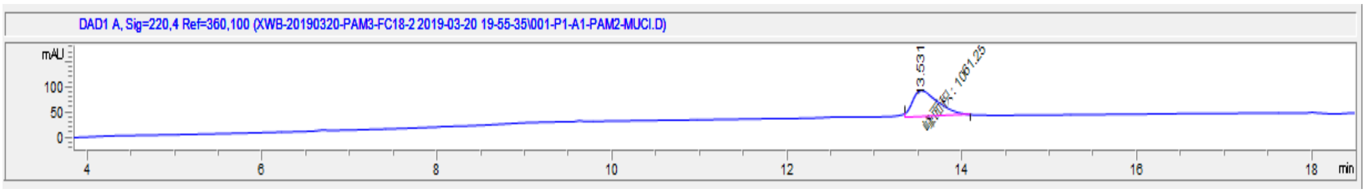

Figure S12. HPLC chromatogram of compound **4**.

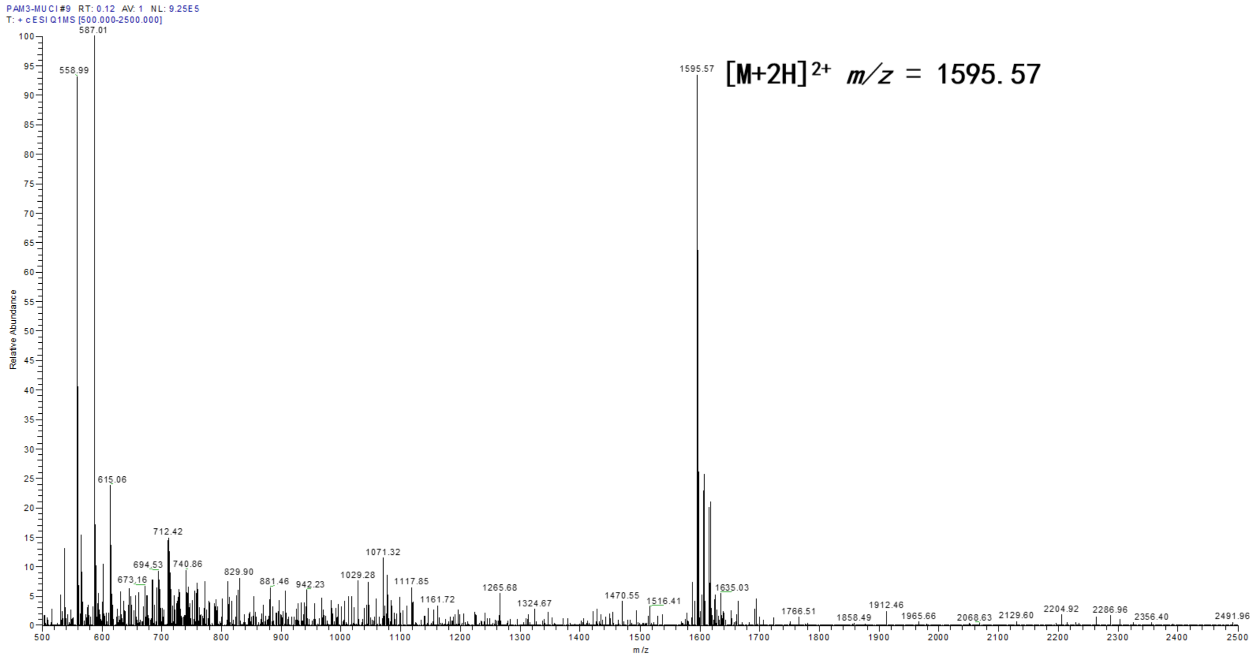

Figure S13. ESI-MS data of compound **4**.

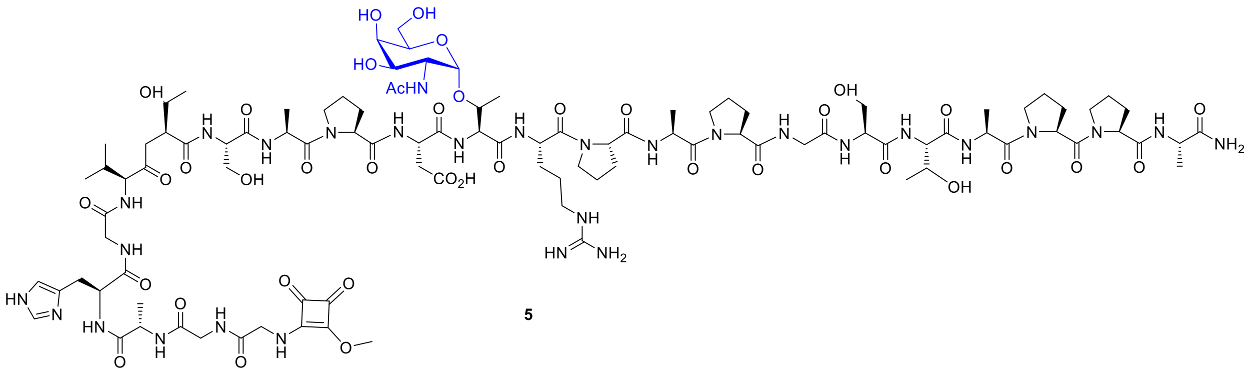

5

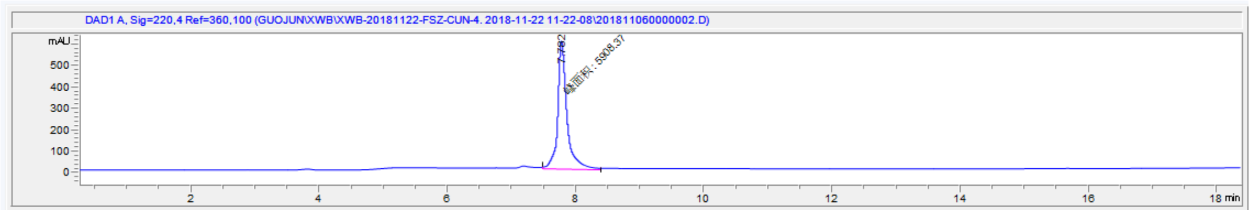

Figure S14. HPLC chromatogram of compound **5**.

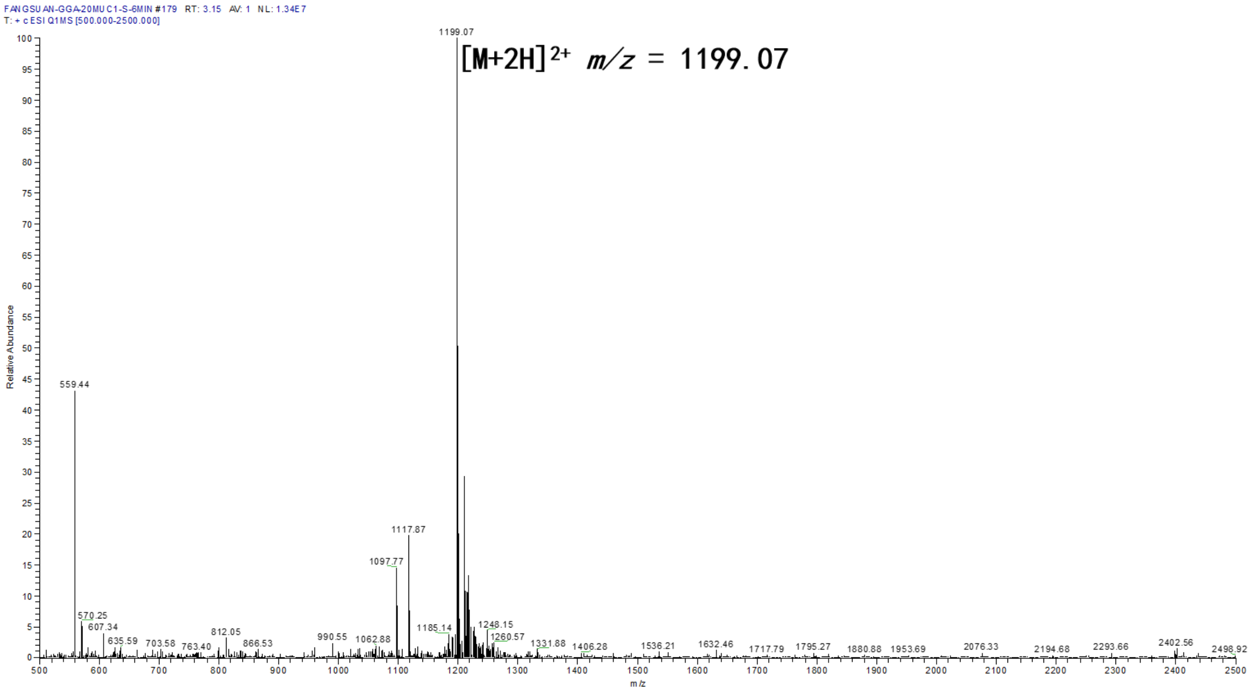

Figure S15. ESI-MS data of compound **5**.

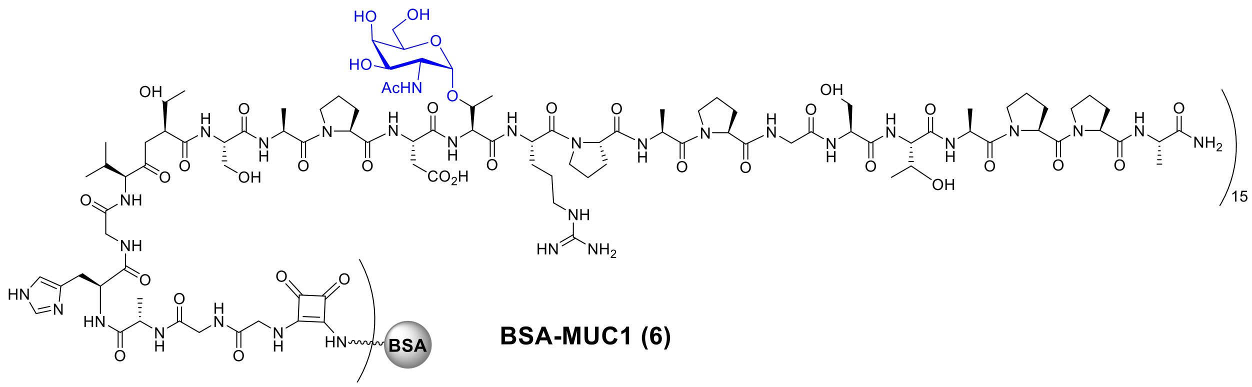

BSA-MUC1 (6)

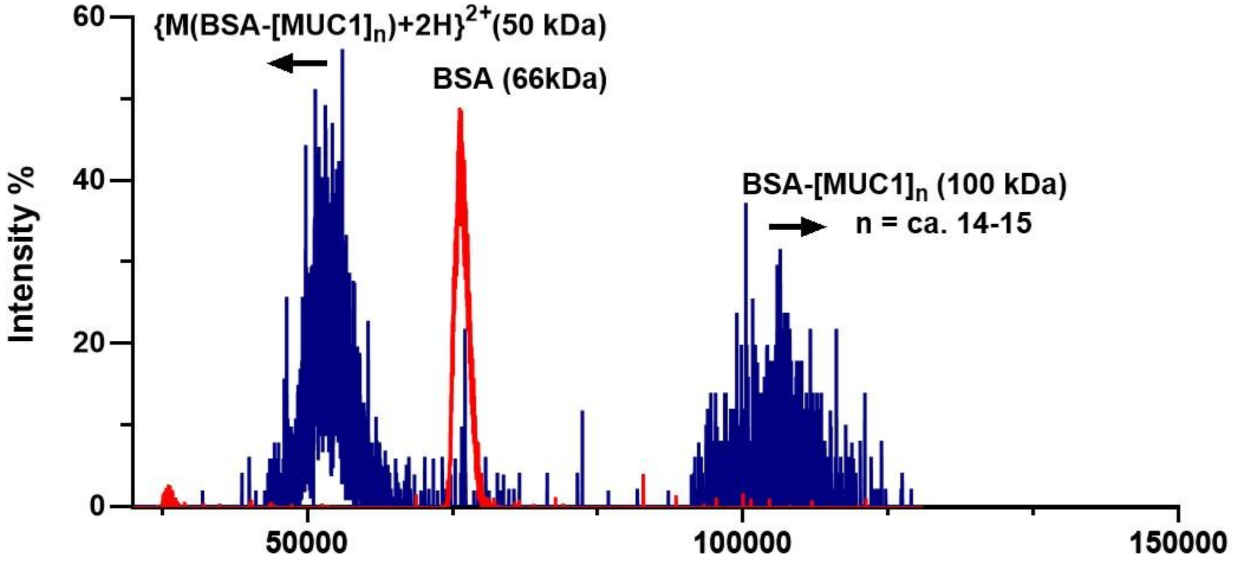

Figure S16. MALDI-TOF-MS data of BSA and BSA-MUC1.
